# Explaining the Decline in Coronary Heart Disease Mortality in the Netherlands between 1997 and 2007

**DOI:** 10.1371/journal.pone.0166139

**Published:** 2016-12-01

**Authors:** Carla Koopman, Ilonca Vaartjes, Ineke van Dis, W. M. Monique Verschuren, Peter Engelfriet, Edith M. Heintjes, Anneke Blokstra, Dorly J. H. Deeg, Marjolein Visser, Michiel L. Bots, Martin O’Flaherty, Simon Capewell

**Affiliations:** 1 Julius Center for Health Sciences and Primary Care, University Medical Center Utrecht, Utrecht, The Netherlands; 2 Dutch Heart Foundation, The Hague, the Netherlands; 3 National Institute for Public Health and the Environment, Bilthoven, the Netherlands; 4 PHARMO Institute for drug research, Utrecht, the Netherlands; 5 EMGO Institute for Health and Care Research, VU Medical Center, Amsterdam, The Netherlands; 6 Department of Health Sciences, VU University, Amsterdam, The Netherlands; 7 Department of Dietetics and Nutrition Sciences, Internal Medicine, VU University Medical Center, Amsterdam, the Netherlands; 8 Department of Public Health & Policy, Institute of Psychology, Health & Society, University of Liverpool, Liverpool, United Kingdom; Department of Cardiology and Angiology, GERMANY

## Abstract

**Objective:**

We set out to determine what proportion of the mortality decline from 1997 to 2007 in coronary heart disease (CHD) in the Netherlands could be attributed to advances in medical treatment and to improvements in population-wide cardiovascular risk factors.

**Methods:**

We used the IMPACT-SEC model. Nationwide information was obtained on changes between 1997 and 2007 in the use of 42 treatments and in cardiovascular risk factor levels in adults, aged 25 or over. The primary outcome was the number of CHD deaths prevented or postponed.

**Results:**

The age-standardized CHD mortality fell by 48% from 269 to 141 per 100.000, with remarkably similar relative declines across socioeconomic groups. This resulted in 11,200 fewer CHD deaths in 2007 than expected. The model was able to explain 72% of the mortality decline. Approximately 37% (95% CI: 10%-80%) of the decline was attributable to changes in acute phase and secondary prevention treatments: the largest contributions came from treating patients in the community with heart failure (11%) or chronic angina (9%). Approximately 36% (24%-67%) was attributable to decreases in risk factors: blood pressure (30%), total cholesterol levels (10%), smoking (5%) and physical inactivity (1%). Ten% more deaths could have been prevented if body mass index and diabetes would not have increased. Overall, these findings did not vary across socioeconomic groups, although within socioeconomic groups the contribution of risk factors differed.

**Conclusion:**

CHD mortality has recently halved in The Netherlands. Equally large contributions have come from the increased use of acute and secondary prevention treatments and from improvements in population risk factors (including primary prevention treatments). Increases in obesity and diabetes represent a major challenge for future prevention policies.

## Introduction

Coronary heart disease (CHD) remains the leading cause of death worldwide and is a major contributor to chronic disease morbidity.[1] Since the 1970s, CHD mortality has fallen dramatically in Western societies. The IMPACT model has been developed to estimate the contribution of changes in uptakes of evidence-based treatments and nationwide changes in cardiovascular risk factors to the changes in CHD mortality. The model has been applied in more than 20 countries.[2–18] Results vary by country, with the contribution from treatments ranging from 25–50% and risk factor changes explaining 50–75%.[2–18] These differences between countries can mostly be explained by the precise time period chosen, and the scale of change in major CHD risk factors.

Analysis from England and then Scotland using an extended IMPACT-SEC model showed that relative inequalities between the most affluent and most deprived groups have actually widened.[4,11] CHD burden tends to differ by socioeconomic circumstances (SEC), with incidence and mortality generally being lower in more affluent groups. Socioeconomic inequalities also exist in the Netherlands, although of smaller magnitude compared to the UK. Nevertheless, the healthy life expectancy without physical limitations is 14 years lower for men with low education compared to men with a high education; for women the difference is 15 years.[19] Furthermore, we have shown previously that socioeconomic inequalities in incidence of acute myocardial infarction (AMI) persisted between 1997 and 2007.[20] Exploration of socioeconomic differences thus remains in the Netherlands.

We therefore aimed to determine what proportion of the recent decline (from 1997 to 2007) in CHD mortality could be attributed to advancements in medical treatment and to nationwide time trends in CHD risk factors, particularly in socioeconomic subgroups of the population.

## Methods

The Dutch population aged 25 years and over between 1997 and 2007 was evaluated using the IMPACT-SEC model.[4,11] This model integrates nationwide data at two time points to explain an observed change in mortality. The IMPACT model was developed to (1) model CHD mortality trends and incorporates time trends in uptake of evidence-based acute phase and secondary prevention treatments, in addition to time trends in major CHD risk factors and (2) estimate the relative change in CHD mortality associated with each of these items. The IMPACT model is validated and applied in several countries.[2–18]

### Data sources

Data used are described in detail in the S1 Appendix. In short, data on the age, sex and socioeconomic distribution of the Dutch population and on specific CHD death counts (International Classification of Diseases version 10 code I20-I25) were obtained from Statistics Netherlands. Dutch inhabitants were divided in three socioeconomic groups: lowest (20% most deprived persons within age-sex-stratum), medium (60% of age-sex-stratum) and highest (20% most affluent persons within age-sex-stratum). Socioeconomic circumstances were based on an area-level socioeconomic indicator constructed by the Netherlands Institute for Social Research in 2002–2006.[21] The national Dutch hospital discharge register was used to determine the number of eligible patients and their 1-year mortality rate. Information on drug use came from the PHARMO Database Network, linking pharmacy and hospitalization records of over 2.3 million subjects.[22–24] Data on drug use in the community came from the general practitioners (GP) register HNU (Huisartsen Netwerk Utrecht). Data of major cardiovascular risk factors (blood pressure, cholesterol, body mass index (BMI), diabetes, smoking, physical inactivity) came from the Doetinchem/MORGEN (RIVM) cohort for those aged up to 65 years and the LASA (Longitudinal Aging Study Amsterdam) cohort for those aged 65 years and over.[25,26] Smoking information came from annual nationwide surveys on smoking habits (STIVORO) and diabetes information from the GP register.

The primary output was the number of Deaths Prevented or Postponed (DPP) in 2007 due to lower CHD mortality rates. The DPP was calculated as the difference between the observed 2007 CHD deaths and the expected CHD deaths in 2007, had 1997 mortality rates remained constant. Change in population size and age distribution was considered using indirect standardization to the Dutch population of 2007. The expected number of CHD deaths was calculated by multiplying age-sex-socioeconomic group-specific mortality rates in 1997 by the population size for each 10-year age-sex-socioeconomic stratum in 2007.

### Deaths prevented or postponed: treatment uptake

For example, in 2007, 1,143 men aged 65 to 74 years in the most deprived group were hospitalized with an acute myocardial infarction (AMI), of whom, 81% received aspirin. Aspirin reduces one year mortality rate by 15% based on recent meta-analyses.[27] The one year mortality rate in these men was 8% in 1997. The approximate number of DPP attributable to aspirin use in AMI for 65–74 year old men in the most deprived group was calculated as:

$$\begin{array}{l} patient\;numbers*treatment\;uptake*relative\;mortality\;reduction\\ \;*1\;year\;mortality\;rate{=1,143*81\%*15\%*8\%\;\approx 11\;DPP}^{} \end{array}$$

As all treatments were in use in 1997, the net benefit of an intervention in 2007 was calculated as:

expectedDPPs(usingtreatmentuptakesin1997)–observedDPPs(usingtreatmentuptakesin2007)

Simply assuming that the efficacy of multiple treatments was additive would overestimate the treatment effect. The Mant and Hicks method was used instead to estimate one-year mortality reduction by polypharmacy.[28] This approach estimates the cumulative relative benefit:

$$\begin{array}{l} relative\;benefit=1-(1-relative\;reduction\;in\;mortality\;rate\;for\;treatment\;A)\\ \;*(1-relative\;reduction\;in\;mortality\;rate\;for\;treatment\;B)\ldots \ldots *(1\\ \;-relative\;reduction\;in\;mortality\;rate\;for\;treatment\;N) \end{array}$$

The acute-phase and secondary prevention treatment component of the model comprised seven mutually exclusive CHD subgroups: patients hospitalized for an AMI, unstable angina, or heart failure associated with CHD and patients in the community who were AMI survivors, or who were patients with stable coronary artery disease (with and without percutaneous or surgical revascularization), or who were patients with CHD-related heart failure. Within each of these groups, 42 medical and surgical therapies were evaluated (listed in Table 1).

**Table 1:** Deaths Prevented or Postponed (DPPs) due to change in acute-phase and secondary prevention treatments for CHD between 1997 and 2007.

		Treatment uptake				
	Nr. of patients	1997 (%)	2007 (%)	RRR (%)	1-year mortality	DPPs, Mean (%) (Range)
**AMI**	18,002				21.0%	668 (6.0) (0.3, 12.3)
Thrombolysis		55.0	2.0	0.24		0
Antiplatelets		87.3	91.0	0.23		29 (0.3) (0.1, 1.1)
B-Blocker		75.9	89.5	0.04		18 (0.2) (-0.2, 1.3)
ACE inhibitor or ARB		29.9	57.4	0.07		45 (0.4) (0.1, 1.1)
Clopidogrel		0.9	77.7	0.03		66 (0.6) (0.1, 2.0)
Primary PCI (within 14 days)		8.0	39.5	0.30		254 (2.3) (0.2, 6.5)
Primary CABG (within 6 wks)		3.8	4.5	0.39		12 (0.1) (0.0, 0.2)
CPR in the community		2.3	4.3	0.79		244 (2.2) (1.8, 2.6)
**Unstable angina**	29,000				7.2%	255 (2.3) (0.4, 6.3)
Heparin		49.6	55.0	0.33		63 (0.6) (0.2, 1.2)
Antiplatelets		76.1	77.1	0.15		18 (0.2) (0.1, 0.4)
IIB/IIIA		0.0	0.6	0.09		0.4 (0.0) (0.00, 0.01)
ACE inhibitor or ARB		17.3	46.1	0.07		40 (0.4) (0.1, 1.0)
B-Blocker		66.8	83.4	0.04		12 (0.1) (-0.1, 0.8)
Clopidogrel		0.1	60.4	0.07		74 (0.7) (0.1, 1.8)
CABG surgery (within 6 wks)		9.4	6.8	0.39		0
PCI (within 14 days)		5.8	14.3	0.32		47 (0.4) (0.0, 1.2)
**Secondary prevention post MI**	110,770				3.9%	228 (2.0) (0.6, 4.8)
Antiplatelets		52.1	52.8	0,15		7 (0.1) (0.0, 0.1)
B-Blocker		40.1	46.6	0.23		61 (0.5) (0.2, 1.3)
ACE inhibitor or ARB		21.8	38.0	0.20		62 (0.6) (0.2, 1.3)
Statin		33.0	47.0	0.24		85 (0.8) (0.2, 1.9)
Acenocoumarol		10.9	10.7	0.22		12 (0.1) (0.0, 0.3)
Rehabilitation		28.5^a^	28.5^a^	0.26		0 (0) (0, 0)
**Secondary prevention post revascularization**	82,467				5.2%	228 (2.0) (0.6, 4.4)
Antiplatelets		51.8	52.7	0.15		6 (0.1) (0.0, 0.1)
B-Blocker		40.4	46.8	0.23		40 (0.4) (0.1, 0.8)
ACE inhibitor or ARB		21.8	38.3	0.20		77 (0.7) (0.2, 1.5)
Statin		33.5	47.6	0.22		101 (0.9) (0.2, 1.8)
Acenocoumarol		11.4	10.8	0.22		3 (0) (0.0, 0.1)
Rehabilitation		28.5^a^	28.5^a^	0.26		0 (0) (0, 0)
**Chronic stable CAD**	277,170				4.0%	1,017 (9.1) (2.6, 18.6)
Antiplatelets		40.4	64.8	0.15		294 (2.6) (1.0, 5.7)
Statins		15.1	50.1	0.23		420 (3.8) (0.8, 7.3)
ACE inhibitor or ARB		16.0	37.9	0.17		303 (2.7) (0.8, 5.5)
CABG surgery (last 5 yrs)		12.1	8.7	0.39		0
**Heart failure in the hospital**	13,320				42.1%	479 (4.3) (1.6, 9.2)
ACE inhibitor or ARB		62.4	71.7	0.20		33 (0.3) (0.1, 0.7)
B-Blocker		22.5	69.2	0.35		388 (3.5) (1.3, 7.3)
Spironolactone		44.9	49.6	0.30		39 (0.4) (0.1, 0.8)
Antiplatelets		45.1	51.0	0.15		19 (0.2) (0.1, 0.4)
**Heart failure in the community**	46,435				18.3%	1,258 (11.2) (4.0, 24.7)
ACE inhibitor/ARB		34.4	59.1	0.20		143 (1.3) (0.4, 2.9)
B-Blocker		19.7	51.9	0.35		566 (5.1) (1.9, 10.7)
Spironolactone		4.5	22.5	0.31		263 (2.3) (0.7, 5.5)
Antiplatelets		31.2	69.1	0.15		287 (2.6) (1.0, 5.6)
**Total treatments**						4,134 (36.9) (10.1, 80.2)

Abbreviations: ACE, angiotensin-converting enzyme; AMI, acute myocardial infarction; ARB, angiotensin II receptor blocker; CABG, coronary artery bypass grafting; CAD, coronary artery disease; CPR, cardiopulmonary resuscitation; PCI, percutaneous coronary intervention (with or without stenting); RRR, relative risk reduction

^a^No change assumed in uptake between 1997 and 2007.

### Deaths prevented or postponed: risk factor changes

Two approaches were used to estimate the number of DPPs due to changes in risk factors. The regression coefficient approach was used for risk factors expressed in continuous data: systolic blood pressure (SBP), total cholesterol level, and BMI. Three variables were used for this approach: (1) the expected number of CHD deaths in 2007, (2) multiplied by the absolute change in risk factor prevalence, (3) multiplied by a regression coefficient that quantified the change in CHD mortality expected for the change in risk factor level. For example, in 2007, there were 161 expected CHD deaths among 202,031 women aged 55 to 64 years in the most deprived group. The mean systolic blood pressure in this group decreased between 1997 and 2007 by 13.3 mm Hg. The relation of blood pressure treatment with CHD mortality estimated age- and sex-specific reduction in CHD mortality to be 50% for every 20-mmHg reduction in SBP, yielding a natural logarithmic coefficient of –0.035.[29] The number of DPP as a result of SBP change was:

$$\begin{array}{c} (1-e^{coefficient\;*\;change})*expected\;deaths\;in\;2007\;=\\ {(1-e}^{-0.035\;*\;13.3})*161\;\approx \;60\;DPP \end{array}$$

The second approach used was the population-attributable risk fraction (PARF). This approach was used to for dichotomous risk factors:

$$PARF=\frac{P*(RR-1)}{1+P*(RR-1)}$$

where P is the risk factor prevalence and RR is the relative risk for CHD mortality associated with the presence of that risk factor. DPP were estimated as the expected CHD deaths in 2007 multiplied by the difference in PARF between 1997 and 2007. For example, diabetes prevalence among men aged 65 to 74 years in the most deprived group was 11% in 1997 and 28% in 2007. Assuming a relative risk of 1.86 constant over time,[30] the PARF was calculated as 0.087 in 1997 and 0.194 in 2007. The number of deaths attributable to the increase in diabetes prevalence from 1997 to 2007 was:

$$\begin{array}{c} expected\;deaths\;in\;2007*(PARF\;in\;2007-PARF\;in\;1997)\;=\\ 887*(0.194-0.087)\approx 95\;additional\;deaths \end{array}$$

### Sensitivity analyses

For each model parameter, minimum and maximum plausible values were assigned using the 95% confidence intervals (from the source documentation); if these were unavailable, these limits were defined as 20% above and below the best estimate. The minimum and maximum plausible values were introduced into the model, generating minimum and maximum estimates for DPP. This represents a conservative estimation of uncertainty as has been applied before.[10,18]

## Results

### Overall findings

Between 1997 and 2007, the age-standardized CHD mortality rate in adults aged 25 years and over fell from 269 to 141 per 100,000 population; a decline of 48% or 6.3% per year (Table 2). In 2007, there were 11,855 CHD deaths, 57% of these were in men. Nationally, there were 11,200 fewer CHD deaths in 2007 than in 1997 mortality rates had persisted, representing the “total” deaths prevented or postponed. A socioeconomic gradient in death rates was observed, with the lowest death rates in the most affluent group. The rate of decline did not differ significantly between socioeconomic groups. Thus, relative socioeconomic inequalities remained stable between 1997 and 2007 (Table 2).

**Table 2:** CHD mortality rates 1997 and 2007 by sex and socioeconomic group.

	Year	National	Most affluent group	Middle group	Most deprived group
		(100%)	(20%)	(60%)	(20%)
**Men**					
Population ≥25 years	1997	5,236,772	890,568	3,237,785	1,108,419
	2007	5,572,741	1,114,194	3,344,676	1,113,871
Observed CHD deaths	1997	11,046	1,644	6,565	2,837
	2007	6,743	1,178	4,014	1,551
Age-standardised rates(per 100,000)^a^	1997	362	316	362	396
	2007	188	167	187	210
Annual % fall^b^		6.3	6.2	6.4	6.1
Expected deaths^c^	2007	13,631	2,342	2,744	3,059
Target DPPs^d^	2007	6,888	1,164	1,405	1,508
% of expected deaths prevented	2007	50.5	49.7	51.2	49.3
**Women**					
Population ≥25 years	1997	5,511,880	936,584	3,391,221	1,184,075
	2007	5,856,439	1,171,650	3,513,791	1,170,998
Observed CHD deaths	1997	8,276	1,327	4,775	2,174
	2007	5,112	889	2,998	1,225
Age-standardised rates(per 100,000)^a^	1997	177	151	175	201
	2007	95	82	93	114
Annual % fall^b^		6.1	5.9	6.2	5.6
Expected deaths^c^	2007	9,423	1,631	1,879	2,157
Target DPPs^d^	2007	4,311	742	879	932
% of expected deaths prevented	2007	45.8	45.5	46.8	43.2
**Total**					
Population ≥25 years	1997	10,748,652	1,827,152	6,629,006	2,292,494
	2007	11,429,180	2,285,844	6,858,467	2,284,869
Observed CHD deaths	1997	19,322	2,971	11,340	5,011
	2007	11,855	2,067	7,012	2,776
Age-standardised rates(per 100,000)^a^	1997	269	234	268	299
	2007	141	125	140	162
Annual % fall^b^		6.3	6.1	6.3	5.9
Expected deaths^c^	2007	23,055	3,972	4,622	5,216
Target DPPs^d^	2007	11,200	1,905	2,285	2,440
% of expected deaths prevented	2007	48.6	48.0	49.4	46.8

^a^ Rates in this table are standardised to the European Standard Population (version 2013) aged 25+ years.

^b^ Annual % fall = (1-(observed 2007 rate/observed 1997 rate)^(1/10)).

^c^ Expected deaths = CHD deaths expected in 2007 based on 2007 population had 1997 CHD rates remained.

^d ^DPPs, deaths prevented or postponed. DPPs = expected – observed deaths in 2007.

As a result of changes in the acute phase and secondary prevention treatment of CHD patients, some 4,134 fewer CHD deaths occurred (Table 3 and Table 1). This accounted for 37% (95% CI: 10% to 80%) of the fall in total CHD mortality in the Netherlands (Table 3). The proportion of changes in uptake of medical and surgical treatment attributable to the fall in CHD deaths was the same across socioeconomic groups. Population level changes in the prevalence of risk factors accounted for 3,979 fewer deaths or 36% (95% CI: 24% to 67%) of the fall (Table 4), of which 25% was related to non-pharmacological changes (in diet and lifestyle) and 11% to changes in the use of blood pressure and cholesterol lowering drugs for primary prevention. Substantial differences existed in the contribution of risk factor changes by sex and socioeconomic circumstances (ranging from 7% in the most affluent women to 57% in the most affluent men, Table 3). However, no consistent gradient or pattern was observed. The model explained 72% of the overall fall in CHD mortality (a shortfall of 3,087 deaths, Table 3). The proportion that could not be explained by changes in treatments and risk factors was larger in the most deprived group (35%) compared to the middle (24%) and most affluent group (29%, Table 3).

**Table 3:** Proportion of CHD deaths prevented or postponed by socioeconomic group, due to change in treatments and risk factors between 1997 and 2007.

	National	Most affluent group	Middle group	Most deprived group
		(100%)	(20%)	(60%)
**Treatments**				
Acute myocardial infarction	6.0%	6.7%	6.0%	5.4%
Unstable angina pectoris	2.3%	2.1%	2.3%	2.5%
Secondary prevention post AMI	2.0%	2.4%	1.8%	2.4%
Secondary prevention post revascularization	2.0%	2.4%	1.8%	2.5%
Chronic stable CAD	9.1%	9.1%	9.0%	9.4%
Heart failure in the hospital	4.3%	1.5%	4.7%	5.4%
Heart failure in the community	11.2%	9.2%	13.0%	7.7%
Total treatments	36.9%	33.3%	38.6%	35.3%
Total treatments – men	32.4%	29.4%	33.3%	32.7%
Total treatments – women	44.1%	38.8%	47.1%	39.4%
**Risk factors**				
Smoking	4.5%	4.0%	4.3%	5.7%
Diabetes	-9.0%	4.5%	-10.0%	-16.5%
Physical inactivity	1.3%	2.9%	1.6%	-0.8%
Systolic blood pressure, mmHg	29.5%	18.4%	31.9%	31.5%
-due to changes in the uptake of blood pressure lowering drugs	3.8%	4.3%	3.8%	3.4%
Total cholesterol, mmol/l	10.4%	9.3%	10.4%	11.2%
-due to changes in the uptake of cholesterol lowering drugs	7.0%	7.8%	6.9%	6.7%
Body Mass Index, m/kg^2^	-1.2%	-1.6%	-1,0%	-1.5%
Total risk factors	35.5%	37.5%	37.1%	29.5%
Total risk factors –men	37.5%	57.0%	34.6%	30.7%
Total risk factors –women	32.4%	7.0%	41.3%	27.6%
**Total treatments + risk factors**				
%DPPs explained by model	72.4%	70.9%	75.7%	64.8%
%DPPs explained by model – men	69.9%	86.3%	67.9%	63.4%
%DPPs explained by model – women	76.6%	45.8%	88.4%	67.0%
%DPPs not explained by model	27.6%	29.1%	24.3%	35.2%
**DPP Counts**				
DPPs explained by the model				
- Due to treatment uptake	4,134 (37%)	635 (33%)	2,639 (39%)	861 (35%)
- Due to risk factor change	3,979 (36%)	715 (38%)	2,544 (37%)	721 (30%)
DPPs unexplained by model	3,087 (28%)	555 (29%)	1,671 (24%)	858 (35%)
Target DPPs	11,200 (100%)	1,905 (100%)	6,854 (100%)	2,440 (100%)

CAD, coronary artery disease. AMI, acute myocardial infarction. DPP, death prevented or postponed.

**Table 4:** Deaths Prevented or Postponed (DPPs) due to changes in risk factors for coronary heart disease *including the effect of changes in primary prevention treatments* between 1997 and 2007.

	Risk factor level	Risk factor level	Absolute change in risk factors^a^	Deaths Prevented or Postponed,
	1997	2007		Mean (%) (range)
**Smoking prevalence**	32.5%	27.2%	-5.3%	507 (4.5) (4.3, 6.5)
**Diabetes prevalence**	5.5%	8.1%	2.6%	-1,003 (-9.0) (-8.3, -12.5)
**Physical inactivity**	60.2%	54.9%	-5.3%	144 (1.3) (1.2, 1.7)
**SBP, mmHg**	132.2	129.4	-2.8	3,304 (29.5) (23.5, 45.8)
	*Treatment uptake*	*Treatment uptake*		
-due to changes in the uptake of blood pressure lowering drugs^**a**^	9.4%	13.7%	+4.3%	422 (3.8) (1.8, 6.8)
**Total cholesterol, mmol/l**	5.6	5.4	-0.2	1,161 (10.4) (7.9, 17.1)
	*Treatment uptake*	*Treatment uptake*		
-due to changes in the uptake of cholesterol lowering drugs^**a**^	0.3%	6.6%	+6.3%	787 (7.0) (1.7, 17.6)
**Body mass index, kg/m**^**2**^	25.9	26.5	0.6	-134 (-1.2) (-0.8, -2.1)
**Total risk factors**				3,979 (35.5) (23.8, 67.0)

SBP, systolic blood pressure.

^a^ Eligible persons (n = 9,747,083) for primary prevention treatment were defined as all persons who did not have a cardiovascular-related hospital admission during the 5 years and 9 months prior to October 1 in the index year, and did not use nitrates, digitalis glycosides or antithrombotic drugs in the index year.[32]

### Changes in acute phase and secondary prevention treatments

The largest contribution to CHD deaths prevented or postponed by increased uptake of treatments came from patients in the community with heart failure (1,258 DPPs, 11%) and chronic stable coronary artery disease (CAD, 1,017 DPPs, 9%), followed by the acute phase treatment of patients with an AMI (668 DPPs, 6%) and hospital treatment of heart failure (470 DPPs, 4%, Table 1). Uptake rates of beta-blockers in heart failure patients in the community more than doubled between 1997 and 2007, representing 5% of the total DPPs. Uptake rates of statins in chronic stable CAD patients increased more than three-fold, representing 4% of the total DPPs. Between 1997 and 2007 small improvements were observed in secondary prevention therapy after a hospital admission for an AMI or revascularization procedure. In general, the improvements due to changes in treatment uptakes were evenly distributed across socioeconomic groups.

### Risk factor changes

The largest contribution from population level changes in risk factors came from the 3 mmHg fall in SBP. This SBP fall generated 3,304 fewer CHD deaths, representing 30% of the CHD mortality decrease (Table 4). Between 1997 and 2007, the uptake of blood pressure lowering drugs for primary prevention therapy increased from 7% to 12% in men and from 11% to 16% in women (Table 4). The pharmacological contribution was fairly small (4% of total DPPs) compared to the non-pharmacological contribution from changes in SBP. The most affluent group showed about one-third smaller total contributions from total SBP changes (19%) compared to the middle and most deprived group (32%). In contrast, the pharmacological benefits were about one-fourth larger in the most affluent (4%) compared to the most deprived (3%) group.

The second largest contribution from risk factor changes was the 0.2 mmol/l fall in cholesterol levels, generating 1,161 (10%) fewer CHD deaths. About two-third of the fall in total cholesterol was related to drug use. The uptake of cholesterol lowering drugs for primary prevention increased between 1997 and 2007 from 0.3% to 7% in men and from 0.2% to 6% in women. In the most affluent SEC group, the largest proportion of DPPs due to changes in cholesterol were related to an increased use of drugs (7.8% pharmacological from 9.3% total, Table 3). Trends in risk factors by SEC group, age and sex are presented in S1 Appendix Table K and L.

Favorable changes in the behavioral risk factors made a modest contribution to DPPs. From 1997 to 2007, smoking prevalence dropped by about 5%, leading to 500 fewer deaths (5% DPPs, Table 4). Socioeconomic gradients were observed in the proportion of DPPs due to changes in smoking and physical inactivity. The most deprived group was the only socioeconomic group that showed increases in physical inactivity, leading to 20 additional deaths (-1% DPPs). The most deprived group, however, did show the largest improvement (-6%) in smoking rates (from 35% to 29%) compared to -4% (from 0% to 26%) in the most affluent group (Table 1, S1 Appendix Table K).

### Adverse risk factor trends

The mortality gains due to positive trends in SBP, cholesterol, smoking and physical inactivity were negated by increases in BMI and diabetes (together contributing 1,137 additional deaths, equivalent to a 10% increase in CHD mortality). The most affluent group was the only SEC group that showed a decline in diabetes (from 6% to 5%, S1 Appendix Table K).

## Discussion

Between 1997 and 2007, Dutch CHD mortality rates fell by an impressive 48%, resulting in 11,200 fewer CHD deaths in 2007. At least 37% of this decline could be attributed to the increased use of acute phase and secondary prevention treatments. Similarly at least 36% could be attributed to changes in population levels of several cardiovascular risk factors (predominantly non-pharmacological). Our results suggest that in the most affluent part of the population the contribution to the decline in CHD mortality was driven by a different set of risk factors changes than in the most deprived part of the population. Approximately 10% more deaths could have been prevented if body mass index and diabetes would not have increased.

### Comparison overall results with other studies

**Fig 1:**
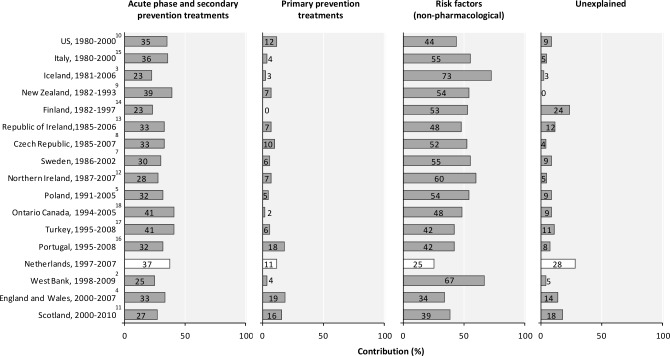
Percentage of the decrease in deaths from coronary heart disease attributed to changes in acute phase and secondary prevention treatments, primary prevention treatments and non-pharmacological risk factor changes in our study population and in other populations. Studies are ranked by starting year.

Several studies reported country specific results from the IMPACT model.[2–18] With respect to the contribution of changes in uptake in medical treatment in the acute phase and in secondary prevention, estimates ranged from 23 to 41%, with our estimate (37%) thus being towards the upper end of the distribution (Fig 1). In all IMPACT studies with a starting year before 1995 non-pharmacological changes in population risk factors explained changes in mortality to a larger extent than in more recently started studies. Our contribution of 25% is the lowest observed. It is to partly reflect our relatively low (3%) non-pharmacological contribution from changes in population cholesterol levels in our study, although the intake of trans fatty acids declined from 4,5% in 1987/1988 to 0.5% in 2007/2010 in the Netherlands.[31] Changes in diet could possibly have had a beneficial effect on CVD mortality beyond the effects of declining cholesterol levels. Some 28% of the mortality fall was not explained by our model. This relatively high percentage may reflect measurement imprecision, lag effects and changes in other, unmeasured risk factor. Although all IMPACT studies have used a broadly similar methodology, the assumptions underlying specific data have inevitably differed between some studies, making direct comparisons imprecise.

### Comparison IMPACT-SEC results with other studies

This is the first IMPACT-SEC study outside the United Kingdom to address socioeconomic differentials concealed within the overall decline in CHD mortality.[4,11] CHD mortality falls were remarkably similar across socioeconomic groups in the Netherlands. This represents a striking contrast with England and Scotland where relative socioeconomic inequalities widened over time.[4,11] Furthermore, in the Netherlands, we also found that the total contribution from risk factor changes was also similar across socioeconomic groups in the Netherlands (although the individual contributions of risk factors differed). The deprived groups benefited from greater falls in smoking rates, blood pressure and cholesterol. However, the most affluent socioeconomic group seemed to show slightly larger benefits from the increased use of blood pressure and cholesterol lowering drugs for primary prevention therapy as compared to the most deprived socioeconomic group. In addition, a more modest change in diabetes prevalence and decline in physical inactivity was observed in the affluent group. This might perhaps reflect more supportive environments, easier access to health care, or greater responsiveness to the general practitioner primary prevention interventions. This insight is very similar to the findings in the UK.[4,11] The underlying contributory factors remain unclear and therefore represent an important area for future investigation.

### Potential implication

Our data highlight several areas meriting greater attention and prevention efforts. For example, the low uptake levels of many secondary preventive medications. Primary prevention interventions should clearly complement secondary prevention efforts, and reflect cost benefit estimates.

### Limitations

The quality of any modelling study is largely determined by the adequacy of the available data to reflect what is going on in a country. Like others we made several assumptions. All were transparent and documented in the S1 Appendix. The use of record linkage represented a substantial methodological improvement compared with some previous IMPACT studies because it allowed accurate accounting for potential overlaps between patient groups. Hospital, population, pharmacy and GP registers were fairly representative for the entire population and large enough for reasonably accurate estimates. Trends in population based risk factor levels were based on individual cohort data, not necessarily precisely representing the entire Dutch population.[25,26] Though not perfect, these remain the best available data for the Netherlands.

Socioeconomic groups were based on an area-level indicator by postal code.[32] Although neighborhood socioeconomic circumstances correlate well with individual socioeconomic position,[33,34] some misclassification may have occurred which could reduce the possibility to observe differences.

### Conclusions

CHD mortality in The Netherlands has dramatically declined since 1997. Equally large contributions have come from the increased use of acute phase and secondary prevention treatments, and from improvements in population risk factors (including primary prevention treatments). These positive trends have been negated by substantial increases in obesity and diabetes, which represent a major challenge for future, more effective prevention policies.

## Supporting Information

S1 Appendix Appendix for the Dutch IMPACT-SEC model, 1997–2007. (PDF)
