# Supplementary material for: Explaining the Decline in Coronary Heart Disease Mortality in the Netherlands between 1997 and 2007
Source: PLoS One. 2016 Dec 1;11(12):e0166139. doi: 10.1371/journal.pone.0166139 (PMC5132334; doi:10.1371/journal.pone.0166139)
Supplement: S1 Appendix — (PDF) [file pone.0166139.s001.pdf]

APPENDIX FOR THE DUTCH IMPACT-SEC MODEL, 1997 – 2007

Explaining the decline in coronary heart disease mortality in the Netherlands between 1997 and 2007 using IMPACT-SEC model. *PLOS ONE 2016*

C Koopman[a,b], I Vaartjes[a,b], I van Dis[b], WMM Verschuren[a,c], P Engelfriet[c], EM Heintjes[d], A Blokstra[c], DJH Deeg[e], M Visser[e,f,g], ML Bots,[a] M O'Flaherty[h], & S Capewell[h]

[a] Julius Center for Health Sciences and Primary Care, University Medical Center Utrecht, Utrecht, The Netherlands.

[b] Dutch Heart Foundation, The Hague, the Netherlands.

[c] National Institute for Public Health and the Environment, Bilthoven, the Netherlands.

[d] PHARMO Institute for drug research, Utrecht, the Netherlands.

[e] EMGO Institute for Health and Care Research, VU Medical Center, Amsterdam, The Netherlands

[f] Department of Health Sciences, VU University, Amsterdam, The Netherlands.

[g] Department of Dietetics and Nutrition Sciences, Internal Medicine, VU University Medical Center, Amsterdam, the Netherlands.

[h] Department of Public Health & Policy, Institute of Psychology, Health & Society, University of Liverpool, Liverpool, UK.

Address correspondence:

Carla Koopman, Julius Center for Health Sciences and Primary Care, University Medical Center Utrecht (STR 6.131), P.O. Box 85500, 3508 GA Utrecht, the Netherlands.

C.Koopman-2@umcutrecht.nl, Telephone: +31 (0) 88 75 68180, Fax: +31 (0) 88 755 5485

Contents	Overview of the IMPACT-SEC model	Page

1	Introduction	3
2	Data sources and examples of deaths prevented or postponed (DPP) calculations	4
2.1	Socioeconomic groups	4
2.2	Changes in mortality rates from CHD, Netherlands 1997 to 2007: Expected and observed number of deaths from CHD	4
2.3	Treatment component of IMPACT-SEC model	4
2.4	Risk factor component of IMPACT-SEC model	6
3	Other methodological considerations	7
3.1	Accounting for poly-pharmacy	7
3.2	Potential overlaps between patient groups: avoiding double counting	9
3.3	Overlap between pharmacological and non-pharmacological contributions to risk factor DPPs	9
3.4	Negative DPPs	10
3.5	Cumulative risk-reduction: adjusting DPPs to calculate cumulative benefit of multiple risk factor changes	10

Table A.	CHD mortality rates in 1997 and 2007 by sex and socioeconomic groups	11
Table B.	Population and patient data sources	12
Table C.	Assumptions and overlap adjustments used in the IMPACT-SEC Model	14
Table D.	Data sources for treatment uptake levels:<br>Medical and surgical treatments included in the model	15
Table E.	Treatment uptake in 1997 and 2007	16
Table F.	Clinical efficacy of interventions: Relative risk reductions obtained from meta-analyses and RCTs	17
Table G.	One-year mortality rates for each patient group	20
Table H.	Risk factors – variable definitions and source	21
Table I.	Beta coefficients for CHD mortality:<br>Systolic blood pressure, total cholesterol, BMI	24
Table J.	Relative risk for CHD mortality:<br>Smoking, diabetes and physical inactivity	25
Table K.	Observed risk factor levels in 1997 and 2007 by sex and socioeconomic group	26
Table L.	Deaths Prevented or Postponed (DPPs) due to changes in risk factors for coronary heart disease including the effect of changes in primary prevention treatments by age and sex between 1997 and 2007	27

	Reference list	28

Overview of the *IMPACT-SEC* model

1. INTRODUCTION

This technical appendix is based on the technical report for the IMPACT-SEC model created using English and Scottish data.[1,2] We have adapted their model to create the Dutch IMPACT-SEC model. However, much of the theory and methods remain. IMPACT is a deterministic, cell-based policy model. The IMPACT model examines the effects of changes in treatment uptake and risk factor trends on changes in mortality from coronary heart disease (CHD) among adults in the Netherlands aged 25 years and over. It uses epidemiological information to estimate the contributions of population-level risk factor changes (impacting mainly on incidence) and changes in the uptake of evidence-based treatments (impacting mainly on case fatality) on mortality decline between two points in time (the start-year and the end-year). The primary outcome measure of the model is the deaths prevented or postponed (DPPs). The extended IMPACT-SEC model accommodates sub-national variation in CHD mortality trends by socioeconomic circumstances (SEC groups). The tables included in this supplementary appendix provide details about the sources and methods that were used in extending the IMPACT model to accommodate socioeconomic circumstances (IMPACT-SEC model).

The starting point for the model is to calculate the 'target' number of CHD deaths the model needs to explain. This target number is obtained by linking the Dutch population register with the Dutch cause of death register to calculate the difference between the actual observed CHD deaths recorded in the end-year and the deaths expected in the end-year had the CHD mortality rates remained the same as in the start-year (i.e. simple direct standardisation).

The calculation of the modelled estimate of DPPs rests on utilising two well-studied relationships: firstly, that between risk factor change and the relative reduction in CHD mortality; secondly, that between treatment uptake and reductions in one-year mortality in patients with a specific form of CHD. The model applies the relative risk reduction quantified in previous randomised controlled trials and meta-analyses to estimate the mortality reduction attributable to: a) temporal change in risk factor prevalence (in those without diagnosed CHD) to calculate the DPPs 'explained' by specific risk factor trends; b) net change over the period in the uptake of specific treatments in patients with each specific form of CHD to estimate DPPs 'explained' owing to improved one-year mortality rates. Great care is taken to avoid double counting of the same individuals.

The mortality benefits from the risk factor reduction in the population, and the treatment benefits in patient groups are then summed. Thus summing uses a cumulative approach (rather than an additive approach), in order to avoid double-counting of benefits in the same individual. (This approach is detailed in Section 3.5). This mortality sum represents the deaths prevented or postponed (DPPs) 'explained' by the model. At the end of the modelling process, the total DPPs 'explained' by the model is then compared with the observed fall in deaths (the 'target' to be explained). Model fit is therefore calculated as the difference between the observed deaths and model DPPs, and expressed as the percentage explained. This measures the extent to which the model was successful in explaining the observed change in CHD mortality in the population.

A policy model like IMPACT thus stands in contrast to a typical multivariate regression model. A typical multivariate regression model represents a statistical approach to describing a single data-set, for instance generated by a single cohort or randomised controlled trial. In contrast, a policy model such as IMPACT seeks to integrate and

synthesise best estimates from a variety of sources to reliably estimate the extent to which a range of factors, acting in combination, explain or predict an outcome. We did not obtain the parameters for this model by running regressions. Rather, the model incorporates the best coefficients from the largest meta-analysis or randomised controlled trials of the reduction in case fatality attributed to treatment or the independent effect sizes of a unit change in each risk factor on CHD mortality.

Examples of the calculation method used for estimating the DPPs due to treatment uptake (Example 1, page 97) and for continuous and binary risk factor change (Examples 2 and 3, respectively, page 98) are provided below. Earlier versions of the IMPACT mortality model have been previously applied to national data from Europe, United States, Ontario, New Zealand and China.[3-7] The methodology has previously been described in detail online and elsewhere.[4-6]

2. DATA SOURCES AND EXAMPLES OF DEATHS PREVENTED OR POSTPONED (DPP) CALCULATIONS

2.1 Socioeconomic groups

We used a socioeconomic indicator by postal code (SCP 2002-2006) as a proxy indicator of socioeconomic circumstances.[8] Socioeconomic scores for a total of 3,965 postal codes were calculated by SCP (Netherlands Institute for Social Research). The mean number of inhabitants was 4,126 per four-digit postal code in 2007. The socioeconomic scores were based on a principal component analysis of the following items: (1) mean annual income per household, (2) percentage of households with low income, (3) percentage of households with low education and (4) percentage of unemployed inhabitants. Rank numbers of socioeconomic scores per postal code were used to make three socioeconomic groups in the Netherlands; lowest socioeconomic group (20% most deprived Dutch inhabitants), middle group (60% of Dutch inhabitants) and highest socioeconomic group (20% most affluent Dutch inhabitants). Socioeconomic circumstances were defined separately in every age-sex stratum. By doing so, the age and sex distribution of the three socioeconomic groups was comparable.

2.2 Changes in mortality rates from CHD, Netherlands 1997 to 2007: Expected and observed number of deaths from CHD

Mortality rates from CHD were calculated using the underlying cause of death (ICD9 code 410-414). Both unadjusted and age-adjusted mortality rates were calculated and presented in Table A. The expected number of CHD deaths in 2007 was calculated by multiplying the age-sex-socioeconomic group specific mortality rates from CHD in 1997 by the population counts for 2007 in that age-sex-socioeconomic stratum. Summing over all strata then yielded the expected number of deaths in 2007 had mortality rates remained unchanged. The difference between the number of expected and observed deaths from CHD represented the mortality fall, or the total number of deaths prevented or postponed (DPP), in 2007 relative to 1997. Population counts, CHD mortality rates, observed and expected numbers of deaths are shown in Table A.

2.3 Treatment component of CHD patients in the IMPACT-SEC model

The treatment component of the IMPACT-SEC model included seven mutually exclusive CHD patient groups (disease group, DG):

DG1. Patients treated in hospital for acute myocardial infarction (AMI, ST-elevation myocardial infarction and non-ST elevation acute coronary syndrome)

DG2. Patients admitted to hospital with unstable angina (UA)
DG3. Community-dwelling patients who have survived a myocardial infarction
DG4. Patients who have undergone a revascularisation procedure: Coronary Artery Bypass Grafting (CABG), or a Percutaneous Coronary Intervention (PCI).
DG5. Community-dwelling patients with stable coronary artery disease (CAD)
DG6. Patients admitted to hospital with heart failure (HF associated with CHD)
DG7. Community-dwelling patients with heart failure (HF associated with CHD)

The data sources used to estimate the size of each disease group (stratified by age-sex-socioeconomic) are shown in Table B. The general approach to calculating the number of DPPs from an intervention among a particular disease group was first to stratify by age, sex and socioeconomic, then to multiply the estimated number of patients in 2007 by the proportion of these patients receiving a particular treatment, by the one-year mortality rate, and by the relative reduction in the mortality rate due to the administered treatment. Sources for treatment uptake are shown in Table D. Sources for estimates of treatment efficacy (relative risk reductions) are shown in Table F. We obtained the relative risks based on the most recent published systematic reviews and meta-analyses of epidemiological studies. Each treatment relative risk value in the model was based on a meta-analysis comparison with an older therapy, or in some cases with a placebo if relevant. Age-sex specific one-year mortality rates for each patient group are presented in Table G. Linked hospital admission and death data were used to calculate historical (1997) one-year mortality rates in the Netherlands where possible (DG1 AMI, DG2 Unstable angina and DG6 Hospital HF). Previously published data[7] was used for the remaining disease groups where Dutch data was not available to calculate rates.

It was assumed that compliance (adherence), i.e. the proportion of treated patients actually taking therapeutically effective levels of medication, was 100% among hospital patients, 70% among symptomatic community patients, and 50% among asymptomatic community patients taking cholesterol-lowering drugs or blood pressure lowering medication for primary prevention. An adjustment was also made in certain cases for sub-optimal dose.

EXAMPLE 1: Estimation of DPPs from a specific treatment
Mortality fall in unstable angina patients as a result of taking aspirin in men aged 65-74 in the most deprived group
For example, in the Netherlands in 2007, about 1143 men aged 65-74 in the most deprived group were hospitalised with unstable angina (ICD-9: 411,413). Uptake of aspirin in this age-sex-socioeconomic stratum was estimated to be approximately 81% in 2007. Aspirin use reduces mortality in patients with unstable angina by approximately 15%. The underlying one-year mortality rate in these men was approximately 8%. The observed DPPs were therefore calculated as:

$$\textit{Patient nrs}_{07} \times \textit{treatment uptake}_{07} \times \textit{relative risk reduction} \times \textit{one-year mortality}$$
$$= 1143 \times 81\% \times 15\% \times 8\% \approx 11 \text{ DPPs}$$

This calculation was then repeated:
a) For all 42 treatments (treatments are listed in the first column of Table E, page 16)
b) For each age-sex-socioeconomic group (70 in total).

c) Incorporating a Mant and Hicks adjustment[9] for multiple medications within each patient group (see Section 3.1).

As all treatments were in use in 1997, the net benefit of an intervention in 2007 was calculated by as: expected DPPs – observed DPPs.

The expected DPPs were calculated as:

Patient nrs$_{07}$ × treatment uptake$_{97}$ × relative risk reduction × one-year mortality

2.4 Risk factor component of IMPACT-SEC model

The second part of the IMPACT-SEC model estimated the number of DPPs related to changes in cardiovascular risk factor levels in the population. The risk factors considered were smoking, total cholesterol, systolic blood pressure, body mass index, diabetes and physical inactivity. Two approaches to calculating DPPs from changes in risk factors were used: the regression approach and change in the Population Attributable Risk Fraction (PARF) approach. These are illustrated below.

Estimating DPPs from risk factor change – regression approach for continuous risk factors

In the regression approach – used for systolic blood pressure (SBP), total cholesterol and body mass index – the number of CHD deaths in 1997 (the start year) after adjusting for population change between 1997 and 2007 were multiplied by the absolute change in risk factor level, and by a regression coefficient ('beta') quantifying the estimated relative change in CHD mortality that would result from a one-unit change in risk factor level (Table I). Natural logarithms were used, as is conventional, in order to best describe the log-linear relationship between absolute changes in risk factor levels and relative change in mortality. Levels of risk factors in 1997 and 2007 by sex and socioeconomic group are shown in Table K.

EXAMPLE 2: Estimation of DPPs from risk factor changes using regression method

Mortality fall due to reduction in SBP in women 55-64 in the most deprived group

For example, in 1997, there were 142 CHD deaths among 178,317 women aged 55-64 years in the most deprived group in the Netherlands. The population total had increased to 202,031 in 2007. Applying the CHD death rate from 1997 (0.8 per 1000) to the 2007 population gives an (adjusted) total of 161 expected deaths in 2007.

Mean SBP in this group fell by an estimated 13.3 millimetres of mercury (mmHg) (from 142.6 in 2000 to 129.3 in 2007). The largest meta-analysis reports an estimated age-sex specific reduction in mortality of 50% for every 20 mmHg reduction in SBP, generating a logarithmic coefficient of -0.035 (i.e. natural logarithm of 0.5 divided by 20). The subsequent reduction in CHD deaths between 1997 and 2007 was then estimated as the product of three variables:

DPPs = expected CHD deaths in 2007 (had 1997 mortality rates remained constant) × absolute risk factor reduction between 1997 and 2007 × regression coefficient exponentiated =

(1-(exponential (regression coefficient × absolute change))) × expected deaths in 2007

= (1-(exponential (-0.035 × 13.3))) × 161 ≈ 60

This calculation was then repeated for each age-sex-socioeconomic group. Data sources for the number of CHD deaths are shown in Table B and data sources for risk factors

trends in Table H. Sources for the regression (beta) coefficients used in these analyses are listed in Table I. The regression coefficients were assumed equal across socioeconomic groups.

Estimating DPPs from risk factor change - PARF approach for binary risk factors
The PARF approach was used for smoking, diabetes, and physical inactivity. PARF, which can be interpreted as the proportion by which the mortality rate from CHD would be reduced if the exposure were eliminated,[10] was calculated as:

$$PARF = [P \times (RR - 1)] / [1 + P \times (RR - 1)]$$

Where P is the prevalence of the risk factor and RR is the relative risk for CHD mortality associated with risk factor presence. A RR of 3.3 associated with smoking, for example, expresses the ratio of risk of CHD mortality in smokers to that in non-smokers. DPPs were then estimated as the expected CHD deaths in 2007 (had 1997 mortality rates remained constant) multiplied by the difference in PARF for 1997 and 2007.

EXAMPLE 3: Estimation of DPPs from risk factor changes using the PARF method
Mortality increase due to increase in diabetes in men aged 65-74 in the most deprived group
For example, the prevalence of diabetes among men aged 65-74 years was 11% in 1997 and 28% in 2007. Assuming a relative risk of 1.86, the PARF for deprived men aged 65-74 was 0.087 in 1998 and 0.194 in 2007.

The DPPs attributable to the increase in diabetes prevalence were therefore:

$$DPPs = \text{expected CHD deaths in 2007 (had 1997 mortality rates remained constant)} \times (PARF_{1997} - PARF_{2007})$$

$$DPPs = 887 \times (0.087 - 0.194) \approx -95 \text{ DPPs}$$

A negative sign for the DPPs denotes deaths increased or brought-forward due to the increase in diabetes prevalence. The calculation was then repeated for each age-sex-socioeconomic group.

Relative risks estimated by expert working groups for the World Health Organization's Global Burden of Disease 2001 Study were used for smoking and physical activity.[11] Effect estimates were based on systematic reviews of cohort studies (adjusted for regression dilution bias) and meta-analyses of randomised controlled trials. Age-variation in the relative risks for diabetes were taken from the DECODE study.[12] These were then applied to the sex-variation in relative risks estimated by Huxley et al.[13] The published relative risk values for smoking, physical activity and diabetes are shown in Table J. These were adjusted in our study to: a) match the 10-year age bands used in IMPACT-SEC and b) employ a dichotomous rather than trichotomous measure of physical activity. Detailed information on how RRs were modified to fit to the age-sex distributions used in the IMPACT-SEC model can be found in the Scottish IMPACT-SEC supplementary appendix (page 41-47).[2] RRs were assumed constant across socioeconomic groups. Age-sex specific RRs are given in Table J.

3. OTHER METHODOLOGICAL CONSIDERATIONS

Other than calculations to take into account change in treatments and risk factors over time, several other adjustments had to be made.

3.1 Accounting for poly-pharmacy

Persons with or at high risk of developing CHD may take a number of different medications. However, data from randomised clinical trials on efficacy of treatment combinations are sparse. Mant and Hicks suggested a method to estimate mortality reduction by poly-pharmacy.[9] The adjustment is carried out in a step-by-step manner as set out in the example below. First the total effect is calculated using an inappropriate additive model, which is then adjusted using effect size calculation with an appropriate multiplicative model.

EXAMPLE 4: Estimation of reduced benefit if patient taking multiple medications (Mant and Hicks approach)

Adjustment for poly-pharmacy in secondary prevention post revascularization in men aged 55-64 in the most affluent group

Taking the example of secondary prevention post myocardial infarction, good evidence (Table F) suggests that, for each intervention, the relative reduction in mortality is approximately: aspirin 15%, beta-blockers 23%, ACE inhibitors (ACE I) 20%, cholesterol lowering drugs 22%, acenocoumarol 22%, and rehabilitation 26%. Our best estimates for uptake were respectively 56%, 46%, 40%, 53%, 10%, and 29%. Assuming a one-year mortality rate of 3% for men aged 55-64 and a total of 3,060 men aged 55-64 residing in the most affluent group in 2007 the total DPPs, with no adjustment for poly-pharmacy, would be calculated as shown in the table below:

Secondary prevention post MI treatment	Nrs	Treatment uptake	Comp-liance	Relative risk reduction	One year mortality rate	Unadjusted DPPs
**Factor**	**A**	**B**	**C**	**D**	**E**	**(A × B × C × D × E)**
**Aspirin**	3,060	56%	70%	15%	3%	5.4
**Beta blockers**	3,060	46%	70%	23%	3%	6.8
**ACE Inhibitors**	3,060	40%	70%	20%	3%	5.1
**Cholesterol lowering drugs**	3,060	53%	50%	22%	3%	5.4
**Acenocoumarol**	3,060	10%	70%	22%	3%	1.4
**Rehabilitation**	3,060	29%	65%	26%	3%	4.5
**Total**						**28.6**

The Mant and Hicks approach suggests that in individual patients receiving all these interventions, mortality reduction is very unlikely to be simply additive. Instead, having considered the 15% mortality reduction achieved by aspirin, the next medication, in this case a beta-blocker, can only reduce the residual mortality (1-0.15). Likewise, the subsequent addition of an ACE inhibitor can then only decrease the remaining mortality, which will be (1-0.15) × (1-0.23). The Mant and Hicks approach therefore suggests that a cumulative relative benefit can be estimated as follows:

Cumulative relative benefit = ***1 – [(1 – (uptake of drug A × relative reduction in mortality rate for drug A)) × (1 – (uptake of drug B × relative reduction in mortality rate for drug B)) × .... × (1 – (uptake of drug N × relative reduction in mortality rate for drug N))]***

In considering appropriate treatments for post revascularization patients, applying relative risk reductions (RRR) for aspirin, beta-blockers, ACE I, cholesterol lowering drugs, acenocoumarol, and rehabilitation then gives the following cumulative relative benefit:

Cumulative relative benefit =1 – [(1 – ($aspirin_{uptake}$ × $aspirin_{RRR}$)) × (1 – (beta $blockers_{uptake}$ × beta $blockers_{RRR}$)) × (1 – (ACE I_{uptake} × ACE I_{RRR})) × (1 – ($statins_{uptake}$ × $statins_{RRR}$)) × (1 – ($warfarin_{uptake}$ × $warfarin_{RRR}$)) × (1 – ($rehabilitation_{uptake}$ × $rehabilitation_{RRR}$)) = 1 – [(1 – (0.56 × 0.15)) × (1 – (0.46 × 0.23)) × (1 – (0.40 × 0.20)) × (1 – (0.53 × 0.22)) × (1 – (0.10 × 0.22)) × (1 – (0.29 × 0.26))] = 1 – [0.92 × 0.89 × 0.92 × 0.88 × 0.98 × 0.92] ≈ 0.40 (i.e. a 40% lower mortality)

Additive benefit = ($aspirin_{uptake}$ × $aspirin_{RRR}$) + (beta $blockers_{uptake}$ × beta $blockers_{RRR}$) + (ACE I_{uptake} × ACE I_{RRR}) + ($statins_{uptake}$ × $statins_{RRR}$)+ ($acenocoumarol_{uptake}$ × $acenocoumarol_{RRR}$) + ($rehabilitation_{uptake}$ × $rehabilitation_{RRR}$))
= (0.56 × 0.15) + (0.46 × 0.23) + (0.40 × 0.20) + (0.53 × 0.22) + (0.10 × 0.22) + (0.29 × 0.26) ≈ 0.48 (i.e. a 48% lower mortality)

This represented a 17% relative reduction 1-(0.40/0.48) on the simple additive value, resulting in 17% fewer DPPs out of an original total of 28.6 DPPs:

Adjusted DPPs = unadjusted DPPs × (cumulative relative benefit / additive benefit) = 28.6 × (0.40/0.48) ≈ 23.8

All treatment DPPs quoted in the results tables refer to the adjusted DPPs.

3.2 Potential overlaps between patient groups: avoiding double counting

To avoid double counting, potential overlaps between different groups of patients were identified and appropriate adjustments made by record linkage or subtracting one group from another. For instance, we can subtract the number of severe heart failure patients treated in hospital from the total number of heart failure patients in the community (because community heart failure patients could be admitted to hospital on one or more occasions). As far as possible record linkage has been used to assign individual patients to only one of the eight disease states; thus avoiding overlaps. A hierarchy of allocation based on mortality was created to assign an individual patient (existing in multiple patient groups) to just one patient group (the one with the highest one-year mortality). The hierarchy structure used was hospitalized HF>hospitalized AMI>hospitalized UA>community HF>community post AMI>community post revascularization. Where this process is not possible then assumptions on overlap adjustment were made showing how potential overlaps were accounted for; these are shown in Table C.

3.3 Pharmacological and non-pharmacological contributions to risk factor DPPs

Risk factor improvements, such as lower blood pressure or total cholesterol, may be achieved through medications, lifestyle changes, or a combination. First we calculated the overall number of DPPs due to changes in mean SBP and total cholesterol levels. Then, we calculated the proportion of DPPs that was due to pharmacological contributions. The estimated effect of blood pressure and cholesterol lowering drugs for primary prevention was calculated in a similar way as treatment effects in the treatment component were calculated (eligible population for primary prevention therapy × treatment uptake × relative risk reduction × one-year mortality rate). All DPPs due to risk factor changes were counted in the risk factor component. The proportion of DPPs

due to pharmacological contributions was presented separately in the risk factor component.

3.4 Negative DPPs (treatments)

In a small number of cases, "negative" DPPs were apparently generated reflecting a decrease in treatment uptake or numbers. For instance with thrombolysis treatments (a larger proportion receiving angioplasty instead of thrombolysis). These negatives were mostly trivial, and were zeroed to reflect the reality: harmful treatments were not being administered. This approach was applied only to disease group (DG)1 AMI in relation to treatment using thrombolysis, DG2 UA in relation to heparin treatment and in DG2 UA and DG5 chronic stable angina in relation to CABG surgery.

3.5 Cumulative risk-reduction: adjusting DPPs to calculate cumulative benefit of multiple risk factor changes

CHD deaths are usually caused by multiple risk factors acting simultaneously. Hence, part of the effect of one risk factor may be mediated through another. For example, physical inactivity may have a direct effect on CHD but may also partly be mediated through its effects on BMI and blood pressure. It is recommended therefore that mortality benefits attributable to risk factors which may be causally related, or which overlap in population groups, should not be combined by simple addition. Ideally, their effects should instead be jointly estimated.[14-18] We do not currently have sources that allow joint estimation of relative risks for combinations of risk factors in this Dutch population. However, several large cohort studies and meta-analyses have published independent risk reduction coefficients for each risk factor included in this study. These are detailed in Tables I and J for continuous and dichotomous risk factors, respectively. One approach commonly used is to calculate the cumulative risk-reduction.[19] This approach accounts for risk factor prevalence overlap but assumes independence of effects.[15,16] The general equation for cumulative risk-reduction is stated as:
Combined (or cumulative) effect = $1 - ((1-a) \times (1-b) \times (1-c) \times \ldots \times (1-n))$

Thus for CHD risk factors, the specific equation is stated as:
$1 - ((1-R_{SBP}) \times (1-R_{smoking}) \times (1-R_{diabetes}) \times \ldots \times (1-R_n))$
where R denotes the mortality change attributable to a specific risk factor.
This is in contrast to additive risk-reduction:
$(R_{SBP})+(R_{smoking})+(R_{diabetes})+\ldots+(R_n)$

The adjustment factor is calculated as: Combined effect/Additive risk-reduction.
The adjustment factor would always be expected to be less than 1. In other words, cumulative risk factor reduction would be smaller than the mortality benefits arrived at by a simple summation of the benefits of each risk factor in turn. In order to avoid positive and negative R values cancelling each other out in the mathematical application, with the perverse effect of the cumulative benefits being apparently greater than the additive in some instances, we first converted all R values into absolute (i.e. sign-free) numbers. We did this on the understanding that the proportional change in CHD mortality associated with risk factor change was independent of the direction of change. The age-sex-socioeconomic adjustment factors fell within the range of 0.78 to 0.97.

TABLE A CHD mortality rates 1997 and 2007 by sex and socioeconomic group

	Year	National	Most affluent group	Middle group	Most deprived group
		(100%)	(20%)	(60%)	(20%)
**Men**					
Population ≥25 years	1997	5,236,772	890,568	3,237,785	1,108,419
	2007	5,572,741	1,114,194	3,344,676	1,113,871
Observed CHD deaths	1997	11,046	1,644	6,565	2,837
	2007	6,743	1,178	4,014	1,551
Age-standardised rates	1997	362	316	362	396
(per 100,000)[a]	2007	188	167	187	210
Annual % fall[b]		6.3	6.2	6.4	6.1
Expected deaths[c]	2007	13,631	2,342	2,744	3,059
Target DPPs[d]	2007	6,888	1,164	1,405	1,508
% of expected deaths prevented	2007	50.5	49.7	51.2	49.3
**Women**					
Population ≥25 years	1997	5,511,880	936,584	3,391,221	1,184,075
	2007	5,856,439	1,171,650	3,513,791	1,170,998
Observed CHD deaths	1997	8,276	1,327	4,775	2,174
	2007	5,112	889	2,998	1,225
Age-standardised rates	1997	177	151	175	201
(per 100,000)[a]	2007	95	82	93	114
Annual % fall[b]		6.1	5.9	6.2	5.6
Expected deaths[c]	2007	9,423	1,631	1,879	2,157
Target DPPs[d]	2007	4,311	742	879	932
% of expected deaths prevented	2007	45.8	45.5	46.8	43.2
**Total**					
Population ≥25 years	1997	10,748,652	1,827,152	6,629,006	2,292,494
	2007	11,429,180	2,285,844	6,858,467	2,284,869
Observed CHD deaths	1997	19,322	2,971	11,340	5,011
	2007	11,855	2,067	7,012	2,776
Age-standardised rates	1997	269	234	268	299
(per 100,000)[a]	2007	141	125	140	162
Annual % fall[b]		6.3	6.1	6.3	5.9
Expected deaths[c]	2007	23,055	3,972	4,622	5,216
Target DPPs[d]	2007	11,200	1,905	2,285	2,440
% of expected deaths prevented	2007	48.6	48.0	49.4	46.8

[a] Rates in this table are standardised to the European Standard Population (version 2013) aged 25+ years

[b] Annual % fall = (1-(observed 2007 rate/observed 1997 rate)^(1/10))

[c] Expected deaths = CHD deaths expected in 2007 based on 2007 population had 1997 CHD rates remained.

[d] DPPs, deaths prevented or postponed. DPPs = expected – observed deaths in 2007

TABLE B Population and patient data sources used in the Dutch IMPACT$_{SEC}$ model

All data by age, sex and socioeconomic group

Information	Source
**Population data**	Record linkage:
Population: counts by age, sex and socioeconomic circumstances	- Dutch population register (inhabitants at Jan 1st of 1997 and 2007)
Deaths: counts by age, sex and socioeconomic circumstances	- Dutch cause of death register (1997 and 2007: primary cause of death ICD-10 I20-I25)
**Number of patients admitted to hospital**	
DG1. Acute myocardial infarction (AMI)	Dutch hospital discharge register (2007: primary diagnosis ICD-9 410)
DG2. Unstable angina pectoris (UA)	Dutch hospital discharge register (2007: primary diagnosis ICD-9 411,413)
DG6. Heart failure (HF)	Dutch hospital discharge register (2007: primary diagnosis ICD-9 428)
**Number of patients in the community eligible for secondary prevention therapies**	
DG3. Post AMI	Record linkage:<br>- Dutch population register (inhabitants Jan 1st 2007)<br>- Dutch hospital discharge register (hospital admission primary diagnosis ICD-9 410 between 1995-2006)
DG4. Post revascularization	Record linkage:<br>- Dutch population register (inhabitants Jan 1st 2007)<br>- Dutch hospital discharge register (main procedure CvV 8837.0, 8837.4, 8837.8, 8837.9 or 5361 between 1995-2006)
DG5. Chronic stable angina (without HF or AMI)	Huisartsen Netwerk Utrecht (HNU):<br>ICPC-code K74 mentioned in the electronic patient record before Jan 1st 2007 without ICPC-code K75 or K77
DG7. Heart failure (HF)	Huisartsen Netwerk Utrecht (HNU, ICPC-code K77 mentioned in the electronic patient record before Jan 1st 2007)
**Number of patients eligible for primary prevention therapies**	
Risk factor component:	Record linkage:
Primary prevention population	- Dutch population register (inhabitants Jan 1st 2007)<br>- Dutch hospital discharge register (exclusion of those with a primary or secondary cardiovascular diagnosis ICD-9 code 401-459 in prior 5 calendar years, exclusion of those with main cardiovascular procedure CvV code 883X 127 535X 536X 537X 538X or 539X in prior 5 calendar years)<br>- PHARMO community pharmacy records (exclusion of those who used nitrates, digitalis glycosides or antithrombotic drugs (ATC code C01DA, C01AA or B01) in 2007)

DG, disease group. ICD, Internation Classification of Diseases code. ICPC, International Classification in Primary Care code. CvV, 'Classificatie van Verrichtingen' code. ATC, Anatomical Therapeutic Chemical classification system

DATA SOURCES POPULATION AND PATIENT NUMBERS

Record linkage in the Netherlands

We linked data between national registers using a record identification number assigned to each resident in the Netherlands with a unique combination of birth date, sex and postal code (about 84% of population). Registries and linking procedures used in this study have been described in detail previously.[20] The quality of the national Dutch registers has been previously investigated – the overall quality is high.[21,22] Linkage of individual data between registers was performed in accordance with the privacy legislation in the Netherlands.

Dutch population register

Data on the size and composition of the Dutch population were provided by the population register. The Netherlands has a population of 16.4 million people in 2007.

Dutch cause of death register

Data on the number of deaths in the Netherlands were derived from the national cause of death register. Primary (one) and secondary (maximum of three) causes of death were coded according to the International Classification of Diseases version 10 (ICD-10). In 1997, 19,322 persons aged 25 years and over died with CHD as primary cause of death. In 2007 this number was 11,855 (-49%).

Dutch hospital discharge register

Hospital discharge records included information concerning primary and secondary diagnoses, performed medical procedures, and dates of hospital admission and discharge. Hospital discharge diagnoses were coded according to ICD-9. Performed medical procedures were coded using 'Classificatie van Verrichtingen' codes. The Dutch hospital discharge register was available electronically from 1995 onwards.

PHARMO

PHARMO is a database network, i.e. a dynamic cohort study of over 1.4 million persons aged 25 years and over, based on a record linkage system containing drug-dispensing records from community and hospital pharmacies linked with hospital discharge records, as previously described.[23] The drug-dispensing records from hospital and community pharmacies contained information concerning the dispensed drug, dispensing date and the prescription length. All prescription drugs were coded according to the Anatomical Therapeutic Chemical (ATC) classification system. Data was available from 1994 onwards.

HNU

'Huisartsen Netwerk Utrecht' (HNU) is a general practitioner (GP) registry of 5 GP practices comprising around 60,000 patients. The registry data we used were collected from 1996 to up to 2013. Information was collected from HIS data (HIS: GP information system).

TABLE C Main assumptions and overlap adjustments used in the IMPACT-SEC model

Treatment category	Assumptions and overlap adjustments	Justification
DG1. Acute myocardial infarction (AMI)	Persons who were already counted in DG6 hospital HF were excluded by record linkage	Record linkage
DG2. Unstable angina pectoris (UA)	Persons who were already counted in DG6 hospital HF or DG1 AMI were excluded by record linkage	Record linkage
DG3. Post AMI	Persons who were already counted in DG6 hospital HF, DG1 AMI or DG2 UA were excluded by record linkage.<br>Furthermore, we assumed that 25% was already counted in DG7 community HF.	Record linkage<br>Weir (2006)[24]
DG4. Post revascularization	Persons who were already counted in DG6 hospital HF, DG1 AMI, DG2 UA or DG3 Post-MI were excluded by record linkage.<br>Furthermore, we assumed that 25% was already counted in DG7 community HF.	Record linkage<br>Weir (2006) [24]
DG5. Chronic stable angina (without HF or AMI)	Those with a history of HF or AMI in the patient record were excluded.<br>Then we deducted the number of persons already counted in DG2 hospital UA.	Capewell (2000)[3]
DG6. Heart failure in hospital associated to CHD	We assumed that 50% of all HF admissions were associated to CHD.	
DG7. Heart failure in the community associated to CHD	We assumed that 50% of all HF patients were associated to CHD.<br>Then we deducted the number of persons already counted in DG6 hospital HF.	
Risk factor component: Population fall in SBP	First, we estimated the overall DPPs from changes in SBP levels. Then, we estimated the number of DPPs that were due to changes in the uptake of blood pressure lowering drugs for primary prevention.	Capewell (1999)[4]<br>Capewell (2000)[3]
Risk factor component: Population fall in total cholesterol	First, we estimated the overall DPPs from changes in total cholesterol levels. Then, we estimated the number of DPPs that were due to changes in the uptake of cholesterol lowering drugs for primary prevention.	

DG, disease group. AMI, acute myocardial infarction. HF, heart failure. CHD, coronary heart disease. DPPs, deaths prevented or postponed. HNU, Huisartsen Netwerk Utrecht. SBP, systolic blood pressure.

TABLE D Data sources for treatment uptake levels

All data by age, sex and socioeconomic group unless otherwise stated

Treatment category	Information	Data source
DG1. Acute myocardial infarction (AMI)	- Drug use during hospital admission (antiplatelets, b-blocker, ACE inhibitor or ARB, clopidogrel)<br>- PCI within 14 days and CABG within 6 weeks of AMI hospital admission<br>- Trombolysis<br>- CPR in the community	- PHARMO record linkage 1998 and 2007<br>- Hospital discharge register 1997 and 2007<br>- Literature (Erasmus MC Rotterdam) 1997, 2007[a,25]<br>- RAVU 2013 for discharge diagnoses of community CPR<br>- ARREST study for uptake CPR[b,26]
DG2. Unstable angina pectoris (UA)	- Drug use during hospital admission (heparin, antiplatelets, IIB/IIIA, b-blocker, ACE inhibitor or ARB, clopidogrel)<br>- PCI within 14 days and CABG within 6 weeks of AMI hospital admission	- PHARMO record linkage 1998 and 2007<br>- Hospital discharge register 1997 and 2007
DG3. Post AMI /DG4. Post revascularization[c]	- Drug use from community pharmacies (antiplatelets, b-blocker, ACE inhibitor or ARB, cholesterol lowering drugs, acenocoumarol)<br>- Rehabilitation	- PHARMO record linkage [c] Drug use 3 years after discharge for AMI in 1998 and 2007<br>- Literature 2007[d,27]
DG5. Chronic stable angina (without HF or AMI)	- Drug use from electronic patient record (antiplatelets, ACE inhibitor or ARB, cholesterol lowering drugs)<br>- CABG surgery (last 5 years)	- HNU 1997 and 2007<br>- Population register Inhabitants Jan 1st 2007<br>- Hospital discharge register CvV code 5361 in 2002-2006 CvV code 5361 in 1995-1996, extrapolated to 1992-1994
DG6. Heart failure in hospital associated to CHD	- Drug use during hospital admission (antiplatelets, ACE inhibitor or ARB, b-blocker, spironolactone)	- PHARMO record linkage 1998 and 2007
DG7. Heart failure in the community associated to CHD	- Drug use from community pharmacies (antiplatelets, ACE inhibitor or ARB, b-blocker, spironolactone)	- HNU 1997 and 2007
Risk factor component: Primary prevention population	- Drug use from community pharmacies (blood pressure and cholesterol lowering drugs)	- PHARMO record linkage 2001-2007 extrapolated to 1997

DG, disease group. HNU, Huisartsen Netwerk Utrecht. RAVU, 'Regionale Ambulance Voorziening Utrecht'.

[a] Data from literature not stratified by age, sex and socioeconomic group. Therefore we applied the age-gradient from English $IMPACT_{SEC}$.

[b] RAVU and ARREST data not stratified by age, sex, socioeconomic group.

[c] Due to small numbers, treatment uptakes estimates for age groups 25-34 yrs and 85+ yrs were not SEC specific, but the average socioeconomic gradient was applied on national values. Furthermore, because of the small numbers and large overlap between patients in DG3 and DG4, treatment uptakes were calculated for DG3 and DG4 combined.

[d] Data on rehabilitation from literature not stratified by age, sex and socioeconomic group and no trend data available. Treatment uptake is assumed equal in 1997 and 2007.

TABLE E Treatment uptake in 1997 and 2007 (For sources see Table D)

List of 42 treatments	National (100%)			Most affluent (20%)			Middle socioeconomic group (60%)			Most deprived (20%)		
	N	Uptake (%)		N	Uptake (%)		N	Uptake (%)		N	Uptake (%)	
		1997	2007		1997	2007		1997	2007		1997	2007
**DG1. Acute myocardial infarction (AMI)**												
Thrombolysis	18002	55,0	2,0	3180	54,6	2,0	10553	55,0	2,0	4268	55,4	2,0
Aspirin	18002	87,3	91,0	3180	91,8	95,9	10553	84,9	91,1	4268	89,8	87,3
B-Blocker	18002	75,9	89,5	3180	67,3	88,8	10553	78,3	89,7	4268	76,4	89,6
ACE I/ARB	18002	29,9	57,4	3180	25,3	51,0	10553	28,5	55,9	4268	36,8	66,0
Clopidogrel	18002	0,9	77,7	3180	0,5	71,7	10553	0,6	78,0	4268	2,2	81,7
Primary PCI	18002	8,0	39,5	3180	8,4	39,0	10553	7,8	39,7	4268	8,2	39,1
Primary CABG	18002	3,8	4,5	3180	3,5	5,3	10553	4,0	4,5	4268	3,4	3,8
Community CPR	18002	2,3	4,3	3180	2,3	4,3	10553	2,3	4,3	4268	2,3	4,3
**DG2. Unstable angina (UA)**												
Heparin	29000	49,6	55,0	5074	38,8	52,5	16999	47,4	56,2	6927	62,8	53,8
Aspirin	29000	76,1	77,1	5074	90,9	75,1	16999	77,3	75,8	6927	62,2	81,9
Platelet glycol-protein IIB/IIIA I	29000	0,0	0,6	5074	0,0	0,7	16999	0,0	0,6	6927	0,0	0,6
ACE I/ARB	29000	17,3	46,1	5074	23,0	49,5	16999	15,3	46,0	6927	18,1	44,0
B-Blocker	29000	66,8	83,4	5074	72,2	78,1	16999	67,4	83,5	6927	61,3	86,8
Clopidogrel	29000	0,1	60,4	5074	0,8	47,2	16999	0,0	63,0	6927	0,0	63,8
CABG (< 6 weeks)	29000	9,4	6,8	5074	9,7	7,6	16999	9,6	7,1	6927	8,7	5,6
PCI (0-14 days)	29000	5,8	14,3	5074	6,1	15,8	16999	6,0	14,4	6927	5,0	12,9
**DG3. Secondary prevention post myocardial infarction**												
Aspirin	110770	52,1	52,8	19491	59,3	54,6	66287	52,9	52,2	24991	44,4	53,2
B-Blocker	110770	40,1	46,6	19491	37,6	45,9	66287	41,0	45,5	24991	39,7	49,9
ACE I/ARB	110770	21,8	38,0	19491	16,4	39,1	66287	22,2	37,4	24991	24,8	38,6
Cholesterol lowering drugs	110770	33,0	47,0	19491	35,7	48,8	66287	34,8	47,0	24991	26,0	45,9
Acenocoumarol	110770	10,9	10,7	19491	9,7	9,7	66287	9,8	10,6	24991	14,7	11,6
Rehabilitation	110770	28,5	28,5	19491	28,5	28,5	66287	28,5	28,5	24991	28,5	28,5
**DG4. Secondary prevention post revascularisation**												
Aspirin	82467	51,8	52,7	15138	57,8	54,0	49244	52,8	52,0	18085	44,2	53,6
B-Blocker	82467	40,4	46,8	15138	37,4	45,7	49244	41,4	45,8	18085	40,4	50,4
ACE I/ARB	82467	21,8	38,3	15138	17,0	39,7	49244	22,1	37,8	18085	25,2	38,7
Cholesterol lowering drugs	82467	33,5	47,6	15138	36,2	49,0	49244	35,7	47,7	18085	25,3	46,2
Acenocoumarol	82467	11,4	10,8	15138	10,7	9,9	49244	10,1	10,9	18085	15,6	11,6
Rehabilitation	82467	28,5	28,5	15138	28,5	28,5	49244	28,5	28,5	18085	28,5	28,5
**DG5. Chronic stable coronary artery disease**												
Aspirin	277170	40,4	64,8	44674	41,4	64,5	170475	36,3	67,0	62022	51,0	58,7
Cholesterol lowering drugs	277170	15,1	50,1	44674	18,8	47,4	170475	14,5	51,9	62022	14,2	47,3
ACE I/ARB	277170	16,0	37,9	44674	23,4	37,6	170475	19,9	37,7	62022	0,0	38,4
CABG surgery (last 5 years)	277170	12,1	8,7	44674	14,7	10,0	170475	11,9	8,6	62022	10,7	8,2
**DG6. Heart failure patients during hospitalisation**												
ACE I/ARB	13320	62,4	71,7	2114	76,3	67,1	8138	58,0	73,6	3068	64,7	69,9
B-Blocker	13320	22,5	69,2	2114	28,5	66,7	8138	21,4	69,2	3068	21,0	70,7
Spironolactone	13320	44,9	49,6	2114	52,0	45,0	8138	44,9	50,2	3068	40,1	50,9
Aspirin	13320	45,1	51,0	2114	45,9	43,3	8138	44,8	51,2	3068	45,3	55,8
**DG7. Heart failurepatients in the community**												
ACE I/ARB	46435	34,4	59,1	6496	48,4	54,7	27998	27,7	61,8	11941	42,7	55,3
B-Blocker	46435	19,7	51,9	6496	13,1	53,5	27998	15,0	52,5	11941	34,3	49,5
Spironolactone	46435	4,5	22,5	6496	8,2	22,2	27998	4,6	22,5	11941	2,3	22,8
Aspirin	46435	31,2	69,1	6496	33,8	71,1	27998	23,3	68,7	11941	48,3	69,0
**Risk factor component. Primary prevention therapies**												
Blood pressure lowering drugs	9747083	9,4	13,7	1949283	8,9	13,1	5849230	9,7	13,8	1948570	9,2	13,8
Cholesterol lowering drugs	9747083	0,3	6,6	1949283	0,3	6,2	5849230	0,4	6,7	1948570	0,0	6,9

TABLE F Clinical efficacy of interventions: relative risk reductions obtained from meta-analyses, and randomised clinical trials

Treatments	Relative risk reduction[a]	Comments	Source paper: First author (year) [ref list], notes
**DG1 Acute myocardial infarction (AMI)**			
**Thrombolysis**	31% (95% CI: 14,45)	<55 years: Odds Ratio (OR)=0.692; Relative Risk Reduction (RRR)=30.8% (95% CI: 14,45)<br>55-64 years: OR=0.736; RRR=26.4% (95% CI: 17,40)<br>65-74 years: OR=0.752; RRR=24.8% (95% CI: 15,37)<br>> 75 years: OR=0.844; RRR=15.6% (95% CI: 4,30)	Estess (2002)[28]
**Aspirin**	23% (95% CI: 15,30)	RRR=23% (95% CI: 15,30): outcome is vascular deaths	ISIS-2 (1988)[29]
**Primary CABG surgery**	39% (95% CI: 23,52)	OR=0.61 (95% CI: 0.48,0.77); RRR=39% (95% CI: 23,52) on page 565, 0-5 year mortality	Yusuf (1994)[30]
**Primary PCI**	30% (95% CI: 15,42)	OR=0.70 (95% CI: 0.58,0.85); RRR=30% (95% CI: 15,42) outcome compares primary angioplasty to thrombolytics.	Keeley (2003)[31]
**Beta blockers**	4% (95% CI: -8,15)	OR=0.96 (95% CI: 0.85,1.08); RRR=4% (95% CI: -8,15) on page 1732	Freemantle (1999)[32]
**ACE inhibitors**	7% (95% CI: 2,11)	OR=0.93 (95% CI: 0.89,0.98); RRR=7% (95% CI: 2,11) for 30 day mortality in myocardial infarction	ACE Inhibitor Myocardial Infarction Collaborative Group (1998)[33]
**Clopidogrel**	3% (95% CI: 1,6)	RRR=3% (95% CI: 1,6) for 30 day mortality in myocardial infarction	Chen (2005)[34]<br>Sabatine (2005)[35]
**Hospital CPR**	33% (95% CI: 10,36)	Survival at 24 hours estimated to be 32%, discharge to home at 21%, and 1 year survival to be 15% overall.	Tunstall-Pedoe (1992)[36]<br>Nadkarni (2006)[37]
**DG2 Unstable angina (UA)**			
**Aspirin**	15% (95% CI: 11,19)	OR=0.85 (95% CI: 0.49,0.95); RRR=15% (95% CI: 11,19). Outcome is vascular and nonvascular deaths on page 75. Assume appropriate for patients with NSTE-ACS.	Antithrombotic Trialists' Collaboration (2002)[38]
**Heparin**	33% (95% CI: -2,56)	OR=0.67 (95% CI: 0.48,1.02); RRR=33% (95% CI: -2,56%) in Table 2. The study outcome is composite MI death and non-fatal MI; compares those on aspirin & heparin to aspirin only.	Oler (1996)[39]
**Platelet glycoprotein IIB/IIA inhibitors**	9% (95% CI: 2,16)	OR=0.91 (95% CI: 0.84,0.98); RRR=9% (95% CI: 2,16). Study looked at acute coronary syndrome without persistent ST elevation.	Boersma (2002)[40]
**Early PCI**	32% (95% CI: 5,51)	OR=0.68 (95% CI: 0.49,0.95); RRR=32% (95% CI: 5,51)	Fox (2005)[41]
**Primary CABG surgery**	39% (95% CI: 23,52)	OR=0.61 (95% CI: 0.48,0.77); RRR=39% (95% CI: 23,52) on page 565, 0-5 year mortality	Yusuf (1994)[30]<br>Assumed similar as STEMI
**Clopidogrel**	7% (95% CI: 2,11)	RRR=7% (95% CI: 2,11)	Yusuf (2001)[42]

**Beta blockers**	4% (95% CI: -8,15)	OR=0.96 (95% CI: 0.85,1.08); RRR=4% (95% CI: -8,15) on page 1732	Freemantle (1999)[32] Assumed similar as STEMI
**ACE inhibitors**	7% (95% CI: 2,11)	OR=0.93 (95% CI: 0.89,0.98); RRR=7% (95% CI: 2,11) for 30 day mortality in myocardial infarction	ACE Inhibitor Myocardial Infarction Collaborative Group (1998)[33]
**DG3/4 Secondary prevention post myocardial infarction/revascularisation**			
**Aspirin**	15% (95% CI: 11,19)	OR=0.85 (95% CI: 0.49,0.95); RRR=15% (95% CI: 11,19). Outcome is vascular and nonvascular deaths on page 75. This data seems to be appropriate to this outcome in CHD patients.	Antithrombotic Trialists' Collaboration (2002)[38]
**Beta blockers**	23% (95% CI: 15,31)	OR=0.77 (95% CI: 0.69,0.85); RRR=23% (95% CI: 15,31) on page 1734. Odds of death in long term trials.	Freemantle (1999)[32]
**ACE inhibitors or Angiotensin-II receptor antagonists**	20% (95% CI: 13,26)	OR=0.80 (95% CI: 0.74,0.87); RRR=20% (95% CI: 13,26) on page 1577, death up to four years [endpoint of study looking at those with heart failure or LV dysfunction].	Flather (2000)[43]
**Cholesterol lowering drugs**	24% (95% CI: 10,26)	RRR=24% (95% CI: 10,26) Intensive statin therapy in acute coronary syndromes.	Hulten (2006)[44]
**Warfarin**	22% (95% CI: 13,31)	OR=0.78 (95% CI: 0.67,0.90); RRR=22% (95% CI: 10,33)	Anand and Yusuf (1999)[45]
**Rehabilitation**	26% (95% CI: 10,39)	OR=0.74 (95% CI: 0.61,0.90); RRR=26% (95% CI: 10,39) in Figure 1, page 685 Taylor reference	Taylor (2004)[46]
**DG5 Chronic stable coronary artery disease**			
**CABG surgery years 0-5**	39% (95% CI:23,52)	OR = 0.61 (95% CI: 0.48-0.77), RRR 39% (95% CI: 23,52) on page 565, 5 year mortality	Yusuf (1994)[30]
**CABG surgery years 6-10**	32% (95% CI: 2,30)	OR = 0.83 (95% CI: 0.70-0.98), RRR 17% (95% CI: 2,30) on page 565, 10 year mortality. OR = 0.68 (95% CI: 0.56-0.83), RRR 32% (95% CI: 17,44) on page 565, 7 year mortality CABG compared to medical treatment	Yusuf (1994)[30]
**Angioplasty**	No effect		Boden (2007)[47]
**Aspirin**	15% (95% CI: 11,19)	OR=0.85 (95% CI: 0.49-0.95); RRR=15% (95% CI: 11,19). Outcome is vascular and nonvascular deaths on page 75.	Antithrombotic Trialists' Collaboration (2002)[38]
**Cholesterol lowering drugs**	23% (95% CI: 10,26)	RRR=23% (95% CI 10,26) Standard dose statin therapy in coronary artery disease.	Wilt (2004)[48]
**ACE inhibitors/ARB**	17% (95% CI: 6,28)	RRR=17% (95% CI 6,28)	Al-Mallah (2006)[49]
**DG6/7 Heart failure in patients requiring hospitalisation or in the community**			
**ACE inhibitors**	20% (95% CI: 13,26)	OR=0.80 (95% CI: 0.74,0.87); RRR=20% (95% CI: 13,26) on page	Flather (2000)[43]

		1577 [death up to four years was study endpoint for those with heart failure or LV dysfunction]	
**Beta blockers**	35% (95% CI: 26,43)	OR=0.65 (95% CI: 0.57,0.74); RRR=35% (95% CI: 26,43): all cause mortality	Shibata (2001)[50]
**Spironolactone**	30% (95% CI: 18,41)<br>31% (95% CI: 18,42)	OR=0.70 (95% CI: 0.59,0.82); RRR=30% (95% CI: 18,41) in those that had at least one cardiac related hospitalisation. OR=0.69 (95% CI: 0.58,0.82); RRR=31% (95% CI: 18,42) in entire study population consisting of those with community heart failure, page 711.	Pitt (1999)[51]
**Aspirin**	15% (95% CI: 11,19)	OR=0.85 (95% CI: 0.49,0.95); RRR=15% (95% CI: 11,19). Outcome is vascular and nonvascular deaths on page 75.	Antithrombotic Trialists' Collaboration (2002)[38]
**Cholesterol lowering drugs**	No effect		Kjekshus (2007)[52]<br>Tavazzi (2008)[53]
**Risk factor component. Primary prevention therapies**			
**Blood pressure lowering drugs**	24% (95% CI:)	RRR=24%;<br>One drug at standard dose with a systolic blood pressure of 150 mmHg and a diastolic blood pressure of 90 mmHg reduces incidence of CHD with 24%, page 12.	Law (2009)[54]
**Cholesterol lowering drugs**	35% (95% CI: 11,52)	OR=0.65 (95% CI: 0.48,0.89); RRR=35% (95% CI: 11,52) for CHD mortality (only trials using statins), Figure 3 on page 4	Pignone (2000)[55]

DG, disease group.

[a]Relative risk reduction (RRR) calculated as 1 – odds ratio

TABLE G One-year mortality rates for each patient group

Patient group (age in years)	DG1[A] Hospitalized AMI	DG2[A] Hospitalized UA	DG6[A] Hospitalized HF	DG3[B] Post AMI	DG4[B] Post revas-cularsation	DG5[B] Chronic stable coronary artery disease	DG7[B] HF in the community	Risk factor component[B] Primary prevention population
**Men**								
**25-34**	0,091	0,029	0,286	0,091	0,250	0,006	0,040	0,000
**35-44**	0,054	0,005	0,200	0,006	0,050	0,009	0,040	0,001
**45-54**	0,046	0,013	0,201	0,006	0,020	0,012	0,060	0,002
**55-64**	0,106	0,035	0,255	0,013	0,030	0,016	0,080	0,006
**65-74**	0,200	0,082	0,361	0,027	0,045	0,029	0,130	0,014
**75-84**	0,366	0,161	0,479	0,067	0,078	0,065	0,200	0,035
**85+**	0,533	0,260	0,603	0,189	0,194	0,163	0,320	0,094
**Women**								
**25-34**	0,150	0,000	0,313	0,008	0,000	0,007	0,050	0,000
**35-44**	0,028	0,005	0,221	0,008	0,000	0,007	0,050	0,001
**45-54**	0,077	0,009	0,168	0,011	0,033	0,010	0,050	0,002
**55-64**	0,120	0,020	0,227	0,014	0,044	0,014	0,080	0,006
**65-74**	0,198	0,052	0,304	0,028	0,064	0,025	0,120	0,014
**75-84**	0,360	0,126	0,402	0,052	0,084	0,054	0,170	0,035
**85+**	0,548	0,241	0,502	0,177	0,083	0,155	0,300	0,094

AMI, acute myocardial infarction. UA, unstable angina. HF, heart failure.

[A] Source: Record linkage Dutch hospital discharge register in 1997 with cause of death register

[B] Source: Ontario Canada, Wijeysundera et.al (2010)[7]

TABLE H Risk factors variable definitions and sources

DATA SOURCES

RIVM

The National Institute for Public Health and the Environment (RIVM) was the main data source for risk factor values in those aged <65 years. We used data from the Doetinchem Cohort Study.[56] The Doetinchem Cohort study started in 1987-1991 (N=7,768, aged 20-59 years at baseline). The study comprised a physical examination for measurements of body weight, height, body mass index (BMI), systolic and diastolic blood pressure, a non-fasting blood sample (total cholesterol and glucose) and several questionnaires about lifestyle and diet. The overall response rate was 62%. Follow-up examinations were carried out every 5years. The response rates for all follow-up measurements varied between 75% and 80%. Blood pressure was measured twice in each examination in sitting position after 2 minutes of rest. The mean value of two measurements was used in the analyses. We used data from examination 2 (1993-1997) and examination 4 (2003-2007). Blood pressure in examination 4 was measured with a different device and participants sat in a slightly different position during the measurement compared with previous examinations. Therefore, blood pressure measurements in examination 4 were statistically adjusted to make blood pressure values in the different examinations comparable.

LASA

The Longitudinal Aging Study Amsterdam (LASA) focuses on physical, emotional, cognitive, and social functioning in older adults. Full details on LASA are provided at www.lasa-vu.nl. In summary, a random sample of older men and women (55-85 years), stratified by age and sex, was drawn from the population registries of 11 municipalities in the Netherlands. Data collection started in 1992/1993 (N=3,107) with participants born between 1908-1937. Further follow-ups were carried out every 3 years since then. In 2002-2003, a new cohort was sampled (birth years 1938-1947, N=1,002) with the same sampling frame as the earlier cohort. Both samples were combined and follow-up was carried out every 3 years (wave). Every examination consists of two parts, a main examination and a medical interview. For the Dutch IMPACT-NL model, data were used from the follow-up wave C 1995/1996 and D 1998/1999 to obtain information for the base year of the model (1997). Follow-up wave F 2005/2006 and G 2008/2009 were used to obtain estimates for the final year in the model (2007). Systolic blood pressure (SBP) was measured once in wave C and D and three times in F and four times in G. Body weight was measured once every wave and corrected for clothes, shoes and corsets. Information on physical activity was obtained during each main interview of LASA.

Risk factor	LASA wave	Description
**SBP (mmHg)**	C, D, F & G	1997: average wave C & wave D<br>2007: average of mean wave F & mean wave G
**Body Mass Index (kg/m^2)**	C, D, F & G	1997: average wave C & wave D<br>2007: average wave F & wave G
**Total cholesterol (mmol/l)**	Only available B,C & G	1997: average wave B & wave C<br>2007: wave G
**Physical inactivity (%)**	C, D, F & G	1997: wave D, if data was missing or if persons

	answered that the activity pattern was not representative for the rest of the year, then wave C was used if that activity pattern was representative<br>2007: wave G, if data was missing or if persons answered that the activity pattern was not representative for the rest of the year, then wave F was used if that activity pattern was representative

Wave B=1992/1993, C=1995/1996, D=1998/1999, F=2005/2006, G=2008/2009

STIVORO
Smoking data was obtained from the Dutch expert centre on tobacco control STIVORO (Stichting Volksgezondheid en Roken, STIVORO, TNS-NIPO Continue Onderzoek Rookgewoonten (COR) 1988-2011). COR annually sent outs a questionnaire to 20,000 adults aged 15 years or over assessing information on the number of people who smoke, the characteristics of smokers and smoking behaviour. In 2011, 26,715 adults were asked to participate and 18,586 (70%) responded. The question in the survey we used was "Do you smoke (sometimes)?" (yes/no). This particular question has not changed during the study period.

HNU
'Huisartsen Netwerk Utrectht' (HNU) is a general practitioner (GP) registry of 5 GP practices comprising around 60,000 patients. The registry data we used were collected from 1996 to up to 2013. Information used from HNU for the IMPACT-NL model was diabetes prevalence, defined as an ICPC-code T90 mentioned in the electronic patient record. Information was collected from HIS data (HIS: GP information system).

RISK FACTORS

Systolic blood pressure
We used RIVM data for the age groups 25-54 years and LASA data for the age group of ≥55 years. For the age group of 25-34 years we had no trend data available, therefore we assumed similar trends as in the age group 35-44 years. Due to the small numbers we used a weighted average for all persons above the age of 75 years. Trends in the age groups 75-84 years and 85+ were assumed equal.

Total Cholesterol
We used RIVM data for the age groups 25-64 years and LASA data for the age group of ≥65 years. For the age group of 25-34 years we had no trend data available, therefore we assumed similar trends as in the age group 35-44 years. In LASA the time period between available cholesterol data from wave C and G was 13 year. Because our trend 1997-2007 was 10 years we applied a 10/13 adjustment factor to the change in total cholesterol.

BMI
We used RIVM data for the age groups 25-64 years and LASA data for the age group of ≥65 years. For the age group of 25-34 years we had no trend data available, therefore we assumed similar trends as in the age group 35-44 years.

Smoking

STIVORO data was provided in 10-year age bands while IMPACT uses 10 year age groups. We adapted the data to fit the IMPACT age groups.
We calculated four separate socioeconomic gradients: RIVM-1997, RIVM-2007, LASA-1997 and LASA-2007. The four socioeconomic gradients were applied to the STIVORO data, which did not have socioeconomic information available.

Physical inactivity

We used RIVM data for the age groups 25-54 years and LASA data for the age group of ≥55 years. For the age group of 25-34 years we had no trend data available, therefore we assumed similar trends as in the age group 35-44 years. Physical inactivity is defined as not complying to the 'Nederlandse Norm Gezond Bewegen'. Adults aged 25–55 years should do at least 30 minutes of vigorous-intensity aerobic physical activity (≥4 metabolic equivalents (METs)) on at least five days in the week. Persons aged ≥55 years should do at least 30 minutes of moderate-intensity (≥3 METs) aerobic physical activity on at least five days in the week.[57]

Diabetes prevalence

We obtained diabetes data from five different sources: RIVM, LASA, CMR-Nijmegen, RNH-Limburg and HNU and noticed substantial differences in trends over time.[58]

Sex	Data source	Trend 1997-2007
**Men**	**RIVM**	- 2%
	**LASA**	+ 114%
	**CMR-Nijmegen**	+ 75%
	**RNH-Limburg**	+ 60%
	**HNU**	+ 252%
**Women**	**RIVM**	- 14%
	**LASA**	+ 119%
	**CMR-Nijmegen**	+ 40%
	**RNH-Limburg**	+ 33%
	**HNU**	+ 248%

Looking at sample size and availability of age-sex-SEC specific data, HNU was the best available data source. Since the information on diabetes mellitus dealt with previous or current diagnosis of diabetes mellitus, the time window for patients in 1997 was shorter than for those in 2007. As a consequence, the estimates of diabetes mellitus prevalence were underestimated in 1997. Therefore, we adjusted the trend based on the average of three data sources. We used age-sex specific adjustment factors. For persons <55 years we used an adjustment-factor for 1997 based on the mean of RIVM, CMR-Nijmegen and RNH-Limburg. For persons ≥55 years we used an adjustment-factor for 1997 based on the mean of LASA, CMR-Nijmegen and RNH-Limburg. For 2007 we used the raw HNU data.

Based on the availability of socioeconomic data, we calculated four separate socioeconomic gradients: RIVM-1997, RIVM-2007, LASA-1997 and LASA-2007. The four socioeconomic gradients were applied to the calculated mean values from the different sources in 1997 and 2007.

TABLE I Beta coefficients: relation of change in risk factors with change in CHD mortality

Beta coefficients were assumed constant across socioeconomic groups.

**Cholesterol**	**Age groups (years)**					
	**25-44**	**45-54**	**55-64**	**65-74**	**75-84**	**85+**
**CHD mortality reduction per 1 mmol/l**						
Men	0.55	0.53	0.36	0.21	0.21	0.21
Women	0.57	0.52	0.35	0.23	0.23	0.23
**Log coefficient**						
**Men**	**-0.799**	**-0.755**	**-0.446**	**-0.236**	**-0.117**	**-0.083**
Minimum	*-0.639*	*-0.604*	*-0.357*	*-0.189*	*-0.093*	*-0.067*
Maximum	*-0.958*	*-0.906*	*-0.536*	*-0.283*	*-0.140*	*-0.100*
**Women**	**-0.844**	**-0.734**	**-0.431**	**-0.261**	**-0.174**	**-0.051**
Minimum	*-0.675*	*-0.587*	*-0.345*	*-0.209*	*-0.139*	*-0.041*
Maximum	*-1.013*	*-0.881*	*-0.517*	*-0.314*	*-0.209*	*-0.062*

Source: Prospective studies collaborative meta-analysis, Lancet 2007[60]
Units: Percentage change in CHD mortality per 1 mmol/l change in total cholesterol

**Body Mass Index (BMI)**	**Age groups (years)**				
	**<45**	**45-59**	**60-69**	**70-79**	**80+**
James et.al (2004):					
Hazard ratio CHD mortality	0.89	0.91	0.95	0.96	0.97
Risk reduction[a] per 1 kg/m^2	0.11	0.09	0.05	0.04	0.03
Age gradient (45-59 as reference)	1.22	**1.00**	0.56	0.44	0.33
Bogers (2006):					
Relative risks, CHD deaths per 5 BMI units (kg/m^2)		**1.16**			
Relative risks per 1 kg/m^2 applying age gradients from James et. al	1.04	1.03	1.02	1.01	1.01
**Log coefficients**	**0.0363**	**0.0297**	**0.0165**	**0.0132**	**0.0099**
Minimum	*0.0255*	*0.0209*	*0.0116*	*0.0093*	*0.0070*
Maximum	*0.0466*	*0.0381*	*0.0212*	*0.0169*	*0.0127*
Source:	Bogers et al (2006)[61], James et al (2004)[62]				
Units:	Percentage change in CHD mortality per 1 kg/m^2 change in BMI				

[a] Risk reduction = 1 – hazard ratio

Systolic blood pressure	Age group (years)				
	25-44	45-54	55-64	65-74	75+
**Men**					
(hazard ratio CHD mortality per 20 mmHg)	0.49	0.49	0.52	0.58	0.65
(log hazard ratio CHD mortality per 1 mmHg)	**-0.036**	**-0.035**	**-0.032**	**-0.027**	**-0.021**
Minimum	*-0.029*	*-0.028*	*-0.026*	*-0.022*	*-0.017*
Maximum	*-0.043*	*-0.042*	*-0.039*	*-0.032*	*-0.025*
**Women**					
(hazard ratio CHD mortality per 20 mmHg)	0.40	0.40	0.49	0.52	0.59
(log hazard ratio CHD mortality per 1 mmHg)	**-0.046**	**-0.046**	**-0.035**	**-0.032**	**-0.026**
Minimum	*-0.037*	*-0.037*	*-0.028*	*-0.026*	*-0.021*
Maximum	*-0.055*	*-0.055*	*-0.042*	*-0.039*	*-0.031*

Source: Prospective studies collaborative meta-analysis, Lancet 2002[59]
Units: Percentage change in CHD mortality per 20 mmHg change in systolic blood pressure

TABLE J Relative risk for CHD mortality: smoking, diabetes and physical inactivity

Relative risks were assumed constant across socioeconomic groups.

	Smoking	Physical inactivity	Diabetes
(age years)	Source: Ezzati (2004)[11]	Source: Ezzati (2004)[11]	Source: Huxley (2006)[13] Roglic (2010)[12]
**Men**			
**25-34**	5.51 (2.47-12.25)	1.50 (1.35-1.67)	4.33 (3.47-5.20)
**35-44**	5.51 (2.47-12.25)	1.50 (1.35-1.67)	3.22 (2.58-3.86)
**45-54**	3.04 (2.66-3.48)	1.50 (1.35-1.67)	2.14 (1.71-2.57)
**55-64**	2.51 (2.22-2.84)	1.50 (1.35-1.67)	1.99 (1.59-2.39)
**65-74**	1.69 (1.52-1.89)	1.44 (1.30-1.61)	1.86 (1.49-2.23)
**75-84**	1.31 (1.11-1.56)	1.32 (1.19-1.47)	1.71 (1.37-2.05)
**85+**	1.05 (0.78-1.43)	1.23 (1.11-1.37)	1.71 (1.37-2.05)
**Women**	2.26 (0.83-6.14)	1.50 (1.35-1.68)	7.55 (6.04-9.06)
**35-44**	2.26 (0.83-6.14)	1.50 (1.35-1.68)	5.63 (4.51-6.76)
**45-54**	3.78 (3.10-4.62)	1.50 (1.35-1.68)	3.81 (3.05-4.57)
**55-64**	3.21 (2.70-3.82)	1.50 (1.35-1.68)	3.12 (2.50-3.74)
**65-74**	2.17 (1.89-2.47)	1.45 (1.30-1.61)	2.55 (2.04-3.06)
**75-84**	1.58 (1.33-1.88)	1.33 (1.20-1.47)	2.36 (1.89-2.83)
**85+**	1.38 (1.08-1.77)	1.24 (1.13-1.37)	2.36 (1.89-2.83)

Detailed information on how RRs were modified to fit to the age-sex distributions used in the IMPACT-SEC model, can be found in the Scottish IMPACT-SEC supplementary appendix (page 41-47)

TABLE K Observed risk factor levels in 1997 and 2007 by age, sex and socioeconomic group

		National (100%)		Most affluent (20%)		Middle group (60%)		Most deprived (20%)	
		1997	2007	1997	2007	1997	2007	1997	2007
**Smoking prevalence, %**		32,5%	27,2%	29,6%	25,6%	32,9%	27,1%	35,2%	29,4%
Men	<65 years	38,8%	33,2%	34,3%	30,2%	39,7%	33,2%	41,0%	35,3%
	≥65 years	28,0%	15,0%	30,8%	18,0%	25,8%	14,0%	35,1%	18,3%
Women	<65 years	32,8%	28,2%	28,9%	25,7%	33,5%	28,3%	34,6%	30,0%
	≥65 years	14,0%	13,0%	15,4%	15,6%	12,9%	12,2%	17,6%	15,9%
**Diabetes prevalence, %**		5,5%	8,1%	6,1%	5,4%	4,9%	8,1%	5,8%	10,7%
Men	<65 years	3,2%	5,2%	3,0%	3,5%	2,7%	5,1%	3,7%	7,0%
	≥65 years	11,5%	21,1%	14,7%	14,1%	10,7%	21,4%	11,1%	28,0%
Women	<65 years	3,7%	4,6%	3,5%	3,1%	3,1%	4,5%	4,2%	6,2%
	≥65 years	14,9%	19,5%	19,1%	13,0%	13,8%	19,8%	14,3%	25,8%
**Physical inactivity, %**		60,2%	54,9%	60,7%	50,7%	52,6%	57,4%	57,6%	58,8%
Men	<65 years	49,6%	47,8%	47,4%	43,5%	39,0%	49,4%	50,7%	52,5%
	≥65 years	78,3%	74,7%	79,8%	71,0%	76,4%	73,7%	81,5%	86,9%
Women	<65 years	58,5%	49,8%	61,7%	45,1%	50,7%	55,7%	48,6%	50,0%
	≥65 years	88,0%	80,9%	87,8%	78,5%	86,7%	78,3%	93,1%	88,2%
**SBP, mmHg**		132,2	129,4	131,8	129,3	132,2	129,3	132,8	130,0
Men	<65 years	130,5	129,3	131,8	129,7	130,4	129,0	129,3	129,8
	≥65 years	152,4	144,3	150,3	142,9	152,6	145,0	154,2	143,6
Women	<65 years	123,5	121,7	123,4	121,3	123,0	121,8	125,1	121,8
	≥65 years	151,9	144,3	145,6	144,1	153,4	143,4	153,9	147,5
**Cholesterol, mmol/L**		5,6	5,4	5,5	5,4	5,6	5,5	5,6	5,4
Men	<65 years	5,6	5,5	5,6	5,4	5,6	5,6	5,6	5,5
	≥65 years	5,4	4,9	5,3	4,8	5,4	5,0	5,4	4,7
Women	<65 years	5,5	5,4	5,5	5,3	5,5	5,4	5,5	5,4
	≥65 years	5,9	5,6	5,8	5,6	5,9	5,5	5,9	5,6
**Body mass index, kg/m^2**		25,9	26,5	25,6	26,2	25,8	26,3	26,0	26,5
Men	<65 years	25,8	26,5	25,6	26,3	25,9	26,5	25,8	26,2
	≥65 years	26,2	27,1	26,3	27,4	26,2	27,0	26,0	27,2
Women	<65 years	25,3	25,8	25,1	25,6	24,9	25,6	25,5	26,0
	≥65 years	27,8	28,0	26,6	27,1	27,9	27,6	28,1	28,4

TABLE L Deaths Prevented or Postponed due to changes in risk factors for coronary heart disease by age and sex, including the effect of changes in primary prevention treatments, between 1997 and 2007

		Risk factor level 1997	Risk factor level 2007	Absolute change in risk factors[a]	Deaths Prevented or Postponed, Mean (%) (range)
**Smoking prevalence**		32.5%	27.2%	-5.3%	507 (4.5) (4.3, 6.5)
Men	<65 years	38.8%	33.2%	-5.6%	97 (0.9) (0.8, 1.2)
	≥65 years	280%	15.0%	-13.0%	372 (3.3) (3.2, 4.7)
Women	<65 years	32.8%	28.2%	-4.6%	9 (0.1) (0.1, 0.1)
	≥65 years	14.0%	13.0%	-1.0%	29 (0.3) (0.3, 0.5)
**Diabetes prevalence**		5.5%	8.1%	2.6%	-1,003 (-9.0) (-8.3, -12.5)
Men	<65 years	3.2%	5.2%	+2.0%	-118 (-1.1) (-1.0, -1.5)
	≥65 years	11.5%	21.1%	+9.6%	-522 (-4.7) (-4.4, -6.6)
Women	<65 years	3.7%	4.6%	+0.9%	-33 (-0.3) (-0.3, -0.4)
	≥65 years	14.9%	19.5%	+4.6%	-330 (-2.9) (-2.7, -4.1)
**Physical inactivity**		60.2%	54.9%	-5.3%	144 (1.3) (1.2, 1.7)
Men	<65 years	49.6%	47.8%	-1.8%	23 (0.2) (0.2, 0.3)
	≥65 years	78.3%	74.7%	-3.6%	55 (0.5) (0.4, 0.6)
Women	<65 years	58.5%	49.8%	-8.7%	-1 (0.0) (0.0, 0.0)
	≥65 years	88.0%	80.9%	-7.1%	67 (0.6) (0.6, 0.8)
**SBP, mmHg**		132.2	129.4	-2.8	3,304 (29.5) (23.5, 45.8)
Men	<65 years	130.5	129.3	-1.2	382 (3.4) (3.1, 5.5)
	≥65 years	152.4	144.3	-8.1	1,580 (14.1) (11.3, 21.8)
Women	<65 years	123.5	121.7	-1.8	165 (1.5) (1.2, 2.2)
	≥65 years	151.9	144.3	-7.6	1,176 (10.5) (7.8, 16.3)
- due to blood pressure lowering drugs[a]		*Treatment uptake 1997:*	*Treatment uptake 2007:*		
		9.4%	13.7%	+4.3%	422 (3.8) (1.8, 6.8)
Men	<65 years	4.8%	8.6%	+3.6%	54 (0.5) (0.2, 0.9)
	≥65 years	22.8%	33.4%	+10.6%	117 (1.0) (0.5, 1.9)
Women	<65 years	7.1%	10.0%	+2.9%	32 (0.3) (0.1, 0.5)
	≥65 years	32.0%	41.3%	+9.3%	219 (2.0) (0.9, 3.5)
**Total cholesterol, mmol/L**		5.6	5.4	-0.2	1,161 (10.4) (7.9, 17.1)
Men	<65 years	5.6	5.5	-0.1	268 (2.4) (1.9. 4.0)
	≥65 years	5.4	4.9	-0.5	576 (5.1) (3.9, 8.5)
Women	<65 years	5.5	5.4	-0.1	42 (0.4) (0.3, 0.6)
	≥65 years	5.9	5.6	-0.3	275 (2.5) (1.8, 4.0)
- due to cholesterol lowering drugs[a]		*Treatment uptake 1997:*	*Treatment uptake 2007:*		
		0.3%	6.6%	+6.3%	787 (7.0) (1.7, 17.6)
Men	<65 years	0.3%	5.5%	+5.2%	109 (1.0) (0.2, 2.1)
	≥65 years	0.2%	16.6%	+16.4%	212 (1.9) (0.5, 4.8)
Women	<65 years	0.3%	4.0%	+3.7%	92 (0.8) (0.2, 1.8)
	≥65 years	0.5%	17.0%	+16.5%	373 (3.3) (0.8, 8.9)
**Body mass index, kg/m^2**		25.9	26.5	0.6	-134 (-1.2) (-0.8, -2.1)
Men	<65 years	25.8	26.5	+0.7	-38 (-0.3) (-0.2, -0.6)
	≥65 years	26.2	27.1	+0.9	-96 (-0.9) (-0.6, -1.5)
Women	<65 years	25.3	25.8	+0.5	-0.4[a] (0.0) (0.0, 0.0)
	≥65 years	27.8	28.0	+0.2	0.4[a] (0.0) (0.0, 0.0)
**Total risk factors**					**3,979 (35.5) (23.8, 67.0)**

SBP, systolic blood pressure.

[a] Eligible persons for primary prevention treatment (n= 9,747,083) were defined as all persons who did not have a cardiovascular-related hospital admission during the 5 years and 9 months prior to October 1 in the index year, and did not use nitrates, digitalis glycosides or antithrombotic drugs in the index year.[23]

[b] Despite an increase in BMI in women (age-standardized to the Dutch population), DPPs are almost 0. Changes in BMI are inconsistent in women: all age groups showed an increase in BMI, except for the age groups 55-64 years and 75-84 years.

REFERENCES

1. Bajekal M, Scholes S, Love H, Hawkins NM, O'Flaherty M, Raine R, et al. Analysing Recent Socioeconomic Trends in Coronary Heart Disease Mortality in England, 2000–2007: A Population Modelling Study. PLoS Medicine. 2012;9:e1001237.
2. Hotchkiss JW, Davies CA, Dundas R, et al. Explaining trends in Scottish coronary heart disease mortality between 2000 and 2010 using IMPACTSEC model: retrospective analysis using routine data. BMJ 2014;348:g1088.
3. Capewell S, Beaglehole R, Seddon M, McMurray J. Explanation for the Decline in Coronary Heart Disease Mortality Rates in Auckland, New Zealand, Between 1982 and 1993. Circulation 2000;102:1511-6.
4. Capewell S, Morrison CE, McMurray JJ. Contribution of modern cardiovascular treatment and risk factor changes to the decline in coronary heart disease mortality in Scotland between 1975 and 1994. Heart 1999;81:380-6.
5. Ford ES, Ajani UA, Croft JB, Critchley JA, Labarthe DR, Kottke TE, et al. Explaining the Decrease in U.S. Deaths from Coronary Disease, 1980–2000. N Engl J Med 2007;356:2388-98.
6. Unal B, Critchley JA, Capewell S. Explaining the decline in coronary heart disease mortality in England and Wales between 1981 and 2000. Circulation 2004;109:1101-7.
7. Wijeysundera HC, Machado, M., Farahati, F. Association of Temporal Trends in Risk Factors and Treatment Uptake With Coronary Heart Disease Mortality, 1994-2005. JAMA 2010;303:1841-7.
8. Knol FA. From high to low; from low to high: the development of social status of neighbourhoods between 1971 and 1995. The Hague: Sociaal Cultureel Planbureau; 1998 [in Dutch]
9. Mant J, Hicks N. Detecting differences in quality of care: The sensitivity of measures of process and outcome in treating acute myocardial infarction. Br Med J 1995;311:793-6.
10. Porta M. A dictionary of epidemiology: Oxford University Press; 2008.
11. Ezatti M, Lopea AD, Rodgers A, Murray CJL, editors. Comparative quantification of risk. Global and regional burden of disease attributable to selected major risk factors. . Geneva: World Health Organization; 2004.
12. Roglic G, Unwin N. Mortality attributable to diabetes: Estimates for the year 2010. Diabetes Res Clin Pract. 2010;87:15-9.
13. Huxley R, Barzi F, Woodward M. Excess risk of fatal coronary heart disease associated with diabetes in men and women: meta-analysis of 37 prospective cohort studies. Br Med J. 2006;332:73-6.
14. Danaei G, Ding EL, Mozaffarian D, Taylor B, Rehm J, Murray CJL, et al. The Preventable Causes of Death in the United States: Comparative Risk Assessment of Dietary, Lifestyle, and Metabolic Risk Factors. PLoS Medicine 2009;6.
15. Dobson AJ, McElduff P, Heller R, Alexander H, Colley P, D'Este K. Changing patterns of coronary heart disease in the hunter region of New South Wales, Australia. J Clin Epidemiol 1999;52:761-71.
16. Taylor R, Dobson A, Mirzaei M. Contribution of changes in risk factors to the decline of coronary heart disease mortality in Australia over three decades. European Journal of Cardiovascular Prevention & Rehabilitation 2006;13:760-8.
17. Tobias M, Taylor R, Yeh L-C, Huang K, Mann S, Sharpe N. Did it fall or was it pushed? The contribution of trends in established risk factors to the decline in premature coronary heart disease mortality in New Zealand. Aust N Z J Public Health 2008;32:117-25.
18. Yusuf S. Two decades of progress in preventing vascular disease. Lancet 2002;360:2-3.
19. Wald NJ, Law MR. A strategy to reduce cardiovascular disease by more than 80%. Br Med J. 2003;326:1419-23.
20. Koopman C, van Oeffelen AA, Bots ML, et al. Neighbourhood socioeconomic inequalities in incidence of acute myocardial infarction: a cohort study quantifying age- and gender-specific differences in relative and absolute terms. BMC Public Health 2012;12:617.
21. Mackenbach JP, Van Duyne WM, Kelson MC. Certification and coding of two underlying causes of death in The Netherlands and other countries of the European Community. J Epidemiol Community Health 1987;41:156-60.
22. Merry AH, Boer JM, Schouten LJ, et al. Validity of coronary heart diseases and heart failure based on hospital discharge and mortality data in the Netherlands using the cardiovascular registry Maastricht cohort study. Eur J Epidemiol 2009;24:237-47.
23. Koopman C, Vaartjes I, Heintjes EM, et al. Persisting gender differences and attenuating age differences in cardiovascular drug use for prevention and treatment of coronary heart disease, 1998-2010. Eur Heart J 2013;34:3198-205.
24. Weir RAP, McMurray JJV, Velazquez EJ. Epidemiology of heart failure and left ventricular systolic dysfunction afteracute myocardial infarction: Prevalence, clinical characteristics, and prognostic importance. Am J Cardiol 2006;97:13F-25F.
25. Nauta ST, Deckers JW, Akkerhuis M, Lenzen M, Simoons ML, van Domburg RT. Changes in Clinical Profile, Treatment, and Mortality in Patients Hospitalised for Acute Myocardial Infarction between 1985 and 2008. PLoS One 2011;6:e26917.
26. SG Beesems, JA Zijlstra, R Stieglis and RW Koster, Reanimatie buiten het ziekenhuis in Noord-Holland en Twente: resultaten ARREST-onderzoek over 2006-2011. In: Koopman C, van Dis I, Visseren FLJ, Vaartjes I, Bots ML. Hart- en vaatziekten in Nederland 2012, cijfers over risicofactoren, ziekte en sterfte. Den Haag: Hartstichting, 2012. [in Dutch]
27. van Engen-Verheul M, de VH, Kemps H, Kraaijenhagen R, de KN, Peek N. Cardiac rehabilitation uptake and its determinants in the Netherlands. Eur J Prev Cardiol 2013;20:349-56.
28. Estess JM, Topol EJ. Fibrinolytic treatment for elderly patients with acute myocardial infarction. Heart. 2002;87:308-11.
29. ISIS-2 (Second international study of infarct survival) collaborative group. Randomised trial of intravenous streptokinase, oral aspirin, both, or neither among 17,187 cases of suspected acute myocardial infarction: ISIS-2. ISIS-2 (Second International Study of Infarct Survival) Collaborative Group. Lancet 1988;2:349-60.
30. Yusuf S, Zucker D, Peduzzi P, Fisher LD, Takaro T, Kennedy JW, et al. Effect of Coronary-Artery Bypass Graft-Surgery on Survival - Overview of 10-Year Results from Randomized Trials by the Coronary-Artery Bypass Graft-Surgery Trialists Collaboration. Lancet 1994;344:563-70.
31. Keeley EC, Boura JA, Grines CL. Primary angioplasty versus intravenous thrombolytic therapy for acute myocardial infarction: a quantitative review of 23 randomised trials. Lancet 2003;361:13-20.
32. Freemantle N, Cleland J, Young P, Mason J, Harrison J. beta Blockade after myocardial infarction: systematic review and meta regression analysis. Br Med J 1999;318:1730-7.

33. ACE Inhibitor Myocardial Infarction Collaborative Group. Indications for ACE inhibitors in the early treatment of acute myocardial infarction: systematic overview of individual data from 100,000 patients in randomized trials. Circulation 1998;97:2202-12.
34. Chen ZM, Jiang LX, Chen YP, Xie JX, Pan HC, Peto R, et al. Addition of clopidogrel to aspirin in 45,852 patients with acute myocardial infarction: randomised placebo-controlled trial. Lancet 2005;366:1607-21.
35. Sabatine MS, Cannon CP, Gibson CM, Lopez-Sendon JL, Montalescot G, Theroux P, et al. Addition of clopidogrel to aspirin and fibrinolytic therapy for myocardial infarction with ST-segment elevation. N Engl J Med 2005;352:1179-89.
36. Tunstall-Pedoe H, Bailey L, Chamberlain DA, Marsden AK, Ward ME, Zideman DA. Survey of 3765 cardiopulmonary resuscitations in British hopsitals (the BRESUS Study): methods and overall results. Br Med J 1992;304:1347-51.
37. Nadkarni VM, Larkin GL, Peberdy MA, Carey SM, Kaye W, Mancini ME, et al. First documented rhythm and clinical outcome from in-hospital cardiac arrest among children and adults. Jama-Journal of the American Medical Association 2006;295:50-7.
38. Antithrombotic Trialists C. Collaborative meta-analysis of randomised trials of antiplatelet therapy for prevention of death, myocardial infarction, and stroke in high risk patients. BMJ 2002;324:71-86.
39. Oler A, Whooley MA, Oler J, Grady D. Adding heparin to aspirin reduces the incidence of myocardial infarction and death in patients with unstable angina - A meta-analysis. Jama-Journal of the American Medical Association 1996;276:811-5.
40. Boersma E, Harrington RA, Moliterno DJ, White H, Theroux P, Van de Werf F, et al. Platelet glycoprotein IIb/IIIa inhibitors in acute coronary syndromes: a meta-analysis of all major randomised clinical trials. Lancet 2002;359:189-98.
41. Fox KAA, Poole-Wilson P, Clayton TC, Henderson RA, Shaw TRD, Wheatley DJ, et al. 5-year outcome of an interventional strategy in non-ST-elevation acute coronary syndrome: the British Heart Foundation RITA 3 randomised trial. Lancet 2005;366:914-20.
42. Yusuf S, Fox KAA, Tognoni G, Mehta SR, Chrolavicius S, Anand S, et al. Effects of clopidogrel in addition to aspirin in patients with acute coronary syndromes without ST-segment elevation. N Engl J Med 2001;345:494-502.
43. Flather MD, Yusuf S, Kober L, Pfeffer M, Hall A, Murray G, et al. Long-term ACE-inhibitor therapy in patients with heart failure or left-ventricular dysfunction: a systematic overview of data from individual patients. Lancet 2000;355:1575-81.
44. Hulten E, Jackson JL, Douglas K, George S, Villines TC. The effect of early, intensive statin therapy on acute coronary syndrome - A meta-analysis of randomized controlled trials. Arch Intern Med 2006;166:1814-21.
45. Anand SS, Yusuf S. Oral anticoagulant therapy in patients with coronary artery disease: A meta-analysis. Jama-Journal of the American Medical Association. 1999;282:2058-67.
46. Taylor RS, Brown A, Ebrahim S, Jolliffe J, Noorani H, Rees K, et al. Exercise-based rehabilitation for patients with coronary heart disease: Systematic review and meta-analysis of randomized controlled trials. Am J Med 2004;116:682-92.
47. Boden WE, O'Rourke RA, Teo KK, Hartigan PM, Maron DJ, Kostuk WJ, et al. Optimal medical therapy with or without PCI for stable coronary disease. N Engl J Med 2007;356:1503-16.
48. Wilt TJ, Bloomfield HE, MacDonald R, Nelson D, Rutks I, Ho M, et al. Effectiveness of statin therapy in adults with coronary heart disease. Arch Intern Med 2004;164:1427-36.
49. Al-Mallah MH, Tleyjeh IM, Abdel-Latif AA, Weaver WD. Anglotensin-converting enzyme inhibitors in coronary artery disease and preserved left ventricular systolic function - A systematic review and meta-analysis of randomized controlled trials. J Am Coll Cardiol 2006;47:1576-83.
50. Shibata MC, Flather MD, Wang DL. Systematic review of the impact of beta blockers on mortality and hospital admissions in heart failure. European Journal of Heart Failure 2001;3:351-7.
51. Pitt B, Zannad F, Remme WJ, Cody R, Castaigne A, Perez A, et al. The effect of spironolactone on morbidity and mortality in patients with severe heart failure. N Engl J Med 1999;341:709-17.
52. Kjekshus J, Apetrei E, Barrios V, Boehm M, Cleland JGF, Cornel JH, et al. Rosuvastatin in older patients with systolic heart failure. N Engl J Med 2007;357:2248-61.
53. Tavazzi L, Maggioni AP, Marchioli R, Barlera S, Franzosi MG, Latini R, et al. Effect of rosuvastatin in patients with chronic heart failure (the GISSI-HF trial): a randomised, double-blind, placebo-controlled trial. Lancet 2008;372:1231-9.
54. Law MR, Morris JK, Wald NJ. Use of blood pressure lowering drugs in the prevention of cardiovascular disease: meta-analysis of 147 randomised trials in the context of expectations from prospective epidemiological studies. BMJ 2009;338:b1665.
55. Pignone M, Phillips C, Mulrow C. Use of lipid lowering drugs for primary prevention of coronary heart disease: meta-analysis of randomised trials. Br Med J 2000;321:983-6.
56. Verschuren WM, Blokstra A, Picavet HS, Smit HA. Cohort profile: the Doetinchem Cohort Study. Int J Epidemiol 2008;37:1236-41.
57. Kemper HGC, Ooijendijk WTM, Stiggelbout M. Consensus over de Nederlandse Norm voor Gezond Bewegen. Tijdschr Soc Gezondheidsz 2000; 78: 180-183.
58. Blokstra A, van Dis I, Verschuren WMM. Factsheet diabetes: feiten en cijfers. Den Haag: Hartstichting. September 2013. [in Dutch]
59. Lewington S, Clarke R, Qizilbash N, Peto R, Collins R, Prospective Studies C. Age-specific relevance of usual blood pressure to vascular mortality: a meta-analysis of individual data for one million adults in 61 prospective studies. Lancet 2002;360:1903-13.
60. Lewington S, Whitlock G, Clarke R, Sherliker P, Emberson J, Halsey J, et al. Blood cholesterol and vascular mortality by age, sex, and blood pressure: a meta-analysis of individual data from 61 prospective studies with 55000 vascular deaths. Lancet. 2007;370:1829-39.
61. Bogers RP, Hoogenveen RT, Boshuizen H, Woodward M, Knekt P, Van Dam RM, et al. Overweight and obesity increase the risk of coronary heart disease: A pooled analysis of 30 prospective studies. Eur J Epidemiol2006;21:107-.
62. James WPT, Jackson-Leach R, Mhrchu CN, Kalamara E, Shayeghi M, Rigby NJ, et al. Overweight and obesity (high body mass index). In: Ezatti M, Lopez AD, Rodgers A, Murray CJL, editors. Comparative quantification of risk Global and regional burden of disease attributable to selected major risk factors. Geneva: World Health Organization; 2004. p. 497-596.
